# Fis Regulates Type III Secretion System by Influencing the Transcription of *exsA* in *Pseudomonas aeruginosa* Strain PA14

**DOI:** 10.3389/fmicb.2017.00669

**Published:** 2017-04-19

**Authors:** Xuan Deng, Mei Li, Xiaolei Pan, Ruiping Zheng, Chang Liu, Fei Chen, Xue Liu, Zhihui Cheng, Shouguang Jin, Weihui Wu

**Affiliations:** ^1^State Key Laboratory of Medicinal Chemical Biology, Key Laboratory of Molecular Microbiology and Technology of the Ministry of Education, Department of Microbiology, College of Life Sciences, Nankai UniversityTianjin, China; ^2^State Key Laboratory of Medicinal Chemical Biology, College of Life Sciences, Nankai UniversityTianjin, China; ^3^Department of Molecular Genetics and Microbiology, College of Medicine, University of FloridaGainesville, FL, USA

**Keywords:** *Pseudomonas aeruginosa*, Fis, type III secretion system, bacterial virulence, *exsA* transcription

## Abstract

Fis is a versatile DNA binding protein in bacteria. It has been demonstrated in multiple bacteria that Fis plays crucial roles in regulating bacterial virulence factors and optimizing bacterial adaptation to various environments. However, the role of Fis in *Pseudomonas aeruginosa* virulence as well as gene regulation remains largely unknown. Here, we found that Fis was required for the virulence of *P. aeruginosa* in a murine acute pneumonia model. Transcriptome analysis revealed that expression of T3SS genes, including master regulator ExsA, was defective in a *fis*::Tn mutant. We further demonstrate that the continuous transcription of *exsC, exsE, exsB*, and *exsA* driven by the *exsC* promoter was required for the activation of T3SS. Fis was found to specifically bind to the *exsB*-*exsA* intergenic region and plays an essential role in the transcription elongation from *exsB* to *exsA*. Therefore, we found a novel role of Fis in the regulation of *exsA* expression.

## Introduction

*Pseudomonas aeruginosa* is a wide-spread Gram-negative opportunistic human pathogen that causes hospital-acquired infections especially in patients with burns, surgical wounds, cancer or cystic fibrosis (Williams et al., 2010; Gellatly and Hancock, 2013). *P. aeruginosa* causes acute and chronic infections by orchestrating the expression of a variety of virulence factors (Turner et al., 2014; Huber et al., 2016), such as type III secretion system (T3SS) (Anantharajah et al., 2016), iron acquisition (Reinhart and Oglesby-Sherrouse, 2016), biofilm formation (Rybtke et al., 2015) etc., and quorum sensing system (QS) dependent virulence factors such as pyocyanin, rhamnolipids, etc., (Goo et al., 2015; Moradali et al., 2017).

The T3SS injectisome of *P. aeruginosa* plays an important role in acute infections (Hauser, 2009). The T3SS is a syringe-like export machine that injects effectors from the bacteria directly into host cell cytosol, influencing cell signaling or leading to cell death (Cornelis, 2006; Sato and Frank, 2011). Expression of the T3SS genes is directly regulated by ExsA, the activity of which is regulated by a partner-switching mechanism (Diaz et al., 2011). Under non-inducing conditions (high Ca^2+^ or the absence of eukaryotic host cells), ExsA is bound by ExsD and remains inactive (Rietsch and Mekalanos, 2006). Under inducing conditions (Ca^2+^ depletion or contact with host cells), ExsE is secreted through the T3SS machinery, releasing its binding partner ExsC. ExsC then binds to ExsD, disrupting the association between ExsD and ExsA. The free ExsA then binds to and activates the promoters of T3SS genes (Hauser, 2009; Anantharajah et al., 2016).

ExsA was identified as the last gene in the operon composed of *exsC, exsE, exsB*, and *exsA* (Diaz et al., 2011). The promoter of *exsC* (P_*exsC*_) is regulated by ExsA (Diaz et al., 2011). A RNA-seq analysis under a T3SS non-inducing condition revealed a gap between the transcripts of *exsB* and *exsA*, indicating an independent promoter of *exsA* (Wurtzel et al., 2012). Recently, Marsden et al identified a Vfr dependent promoter of *exsA* (P_*exsA*_) located at the *exsB*-*exsA* intergenic region, which regulates *exsA* expression independent of ExsA (Marsden et al., 2016). However, it remains elusive how the *exsC* and *exsA* promoters coordinately regulate the transcription of *exsA* under T3SS inducing conditions.

Fis is an abundant bacterial nucleoid-associated protein, which influences DNA topology by directly binding and bending DNA (Dillon and Dorman, 2010). It also indirectly affects DNA topological state and supercoiling by regulating DNA gyrase and topoisomerase I genes expression (Travers and Muskhelishvili, 2005). Besides, binding of Fis to DNA can introduce an interwound and branched structure in the DNA which may influence the local transcriptional activity (Schneider et al., 2001; Kahramanoglou et al., 2011). It has been demonstrated that Fis coordinates expression of multiple virulence factors in various bacterial pathogens, such as pathogenic *Escherichia coli* (Falconi et al., 2001; Goldberg et al., 2001), *Salmonella enterica serovar* Typhimurium (Schechter et al., 2003; Kelly et al., 2004), *Shigella flexneri* (Falconi et al., 2001), *Dickeya dadantii* (Lautier and Nasser, 2007), and *Yersinia pseudotuberculosis* (Green et al., 2016). *S. enterica* harbors two distinct T3SSs, namely SPI-1 and SPI-2 T3SS, which play important roles in bacterial invasion of nonphagocytic cells and replication inside host cells, respectively (Ellermeier and Slauch, 2007; Figueira and Holden, 2012). It has been found that Fis regulates the SPI-1 and SPI-2 T3SSs through regulatory genes *hilA* and *ssrB*, respectively (Kelly et al., 2004; Wang et al., 2013). In *D. dadantii*, Fis regulates the T3SS through activation of *hrpN* (effector of T3SS) (Lautier and Nasser, 2007). In *E. coli*, the Fis binding sequence has been characterized as GNNBNwwwwwNVNNC (B = not A, V = not T, w = high proportion A or T, and N = any nucleotide) (Cho et al., 2008; Shao et al., 2008; Kahramanoglou et al., 2011; Hancock et al., 2016). In a previous study in *P. aeruginosa*, a fis::Tn mutant was found to be defective in swarming motility (Liberati et al., 2006; Yeung et al., 2009). Meanwhile, the swimming and twitching motility as well as biofilm formation are normal in the *fis*::Tn mutant (Yeung et al., 2009). However, the role of Fis in *P. aeruginosa* virulence as well as its DNA binding sequence has not been well defined.

Here we report that Fis is essential for *P. aeruginosa* virulence in a murine acute pneumonia model and the expression of T3SS genes is defective in a *fis*::Tn mutant. We demonstrate that under T3SS inducing condition, transcription of *exsA* is driven by the P_*exsC*_ and binding of Fis to the intergenic region between *exsB* and *exsA* is essential for the continuation of transcription from *exsB* to *exsA*, which is required for the expression of *exsA*. Therefore, our results reveal a novel role of Fis in the regulation of *exsA* expression.

## Materials and methods

### Bacterial strains and plasmids

The bacterial strains, plasmids and primers used in this study are listed in Table S1. *E. coli* DH5α (TransGen, Beijing, China), S17-1 (Simon et al., 1983) strains used for general cloning conjugal transferring (Chen et al., 2016) were cultured in Luria–Bertani (LB) broth [10 g/l tryptone (Oxoid Ltd., Basingstoke, UK), 5 g/l NaCl (BBI life sciences, Shanghai, China), 5 g/l yeast extract (Oxoid Ltd.), pH 7.0–7.5] or LB agar (LB broth containing 15 g/l agar) under aerobic conditions at 37°C. When needed, the medium was supplemented with tetracycline (50 μg/ml) (BBI life sciences), gentamicin (100 μg/ml) (BBI life sciences), or carbenicillin (150 μg/ml) (BBI life sciences) for *P. aeruginosa* PA14 (Liberati et al., 2006), and ampicillin (100 μg/ml) (BBI life sciences) or tetracycline (10 μg/ml) for *E. coli*. When needed, Isopropyl β-D-1-thiogalactopyranoside (IPTG) at indicated concentrations was added to culture mediums.

For DNA manipulation, standard protocols or manufacture instructions of commercial products were followed. For the complementation of the *fis* gene, the open reading frame of *fis* and its upstream 486 bp region was amplified by PCR with PA14 chromosomal DNA as the template. The PCR product was cloned into the *Bam*HI-*Eco*RI sites of pUC18T-mini-Tn7T-Tc (Choi and Schweizer, 2006), resulting in pUC18T-mini-Tn7T-Tc-*fis*. The pEX18Tc-T0T1 insertion was constructed by cloning the 934 bp upstream and 987 bp downstream fragments of the *exsB-exsA* intergenic region into the *Eco*RI-*Hin*dIII sites of plasmid pEX18Tc (Hoang et al., 1998), and a 289 bp DNA fragment containing terminators T0T1 was amplified by PCR from pUC18T-mini-Tn7T (Choi and Schweizer, 2006) and inserted in between the two fragments. Chromosomal gene mutations were generated by homologous recombination as described previously (Hoang et al., 1998). To construct the C-terminus His-tagged ExsA driven by its native promoter, a DNA fragment containing the 300 bp fragment upstream of *exsA* and the *exsA* coding region was amplified by PCR, the His-tag coding sequence was included in one of the PCR primers. The PCR product was cloned into the *Xba*I-*Hin*dIII sites of a promoterless pUCP20 (Li et al., 2016). To construct the *exsA* promoter *lacZ* transcriptional fusion (P_*exsA*_-*lacZ*), the 500 bp fragment upstream of the *exsA* coding region was amplified by PCR and cloned into the *Bam*HI-*Eco*RI sites of pDN19*lacZ*Ω. Sequences of the PCR primers were listed in Table S1.

### Real-time PCR

Bacteria were grown at 37°C under indicated conditions to indicated optical density of 600 nm (OD_600_). Total RNA was isolated with a RNAprep Pure Bacteria Kit (Tiangen Biotech, Beijing, China). The cDNA was synthesized from total RNA using random primers and PrimeScript Reverse Transcriptase (TaKaRa, Dalian, China). Specific Primers (Table S1) were used for reverse transcription (RT) and quantitative PCR. For quantitative PCR, cDNA was mixed with 4 pmol of forward and reverse primers and SYBR Premix Ex Taq™ II (TaKaRa) in a total reaction volume of 20 μl. The 30S ribosomal protein coding gene *rpsL* was used as an internal control (Li et al., 2013). The results were determined using a CFX Connect Real-Time system (Bio-Rad, USA).

### Transcriptome sequencing and analysis

The transcriptome sequencing analysis was performed by Beijing Genomics Institute. Total RNA was isolated from bacteria at OD_600_ of 1.0 with an RNAprep Pure Bacteria Kit (Tiangen). After DNase I (NEB) digestion, rRNA was removed from the total RNA by using Ribo-Zero Magnetic Kit (Bacteria, EPICENTRE). The mRNA was fragmented into short fragments by using fragmentation buffer (Ambion). Then cDNA was synthesized using the mRNA fragments as templates. The purified fragmented cDNA was combined with End Repair Mix and A-Tailing Mix for end reparation and single nucleotide A (adenine) addition. Then, the short fragments were connected with adapters. After agarose gel electrophoresis, the suitable fragments were selected for the PCR amplification as templates. During the quality control steps, Agilent 2100 Bioanaylzer and ABI StepOnePlus Real-Time PCR System were used in quantification and qualification of the sample library. At last, the library was sequenced using Illumina HiSeq™ 2000 or other sequencer when necessary. The RNA expression analysis was based on the predicted genes of strain PA14 (http://www.pseudomonas.com). *P*-values were calculated referring to “the significance of digital gene expression profiles” (Audic and Claverie, 1997). A strict algorithm to identify differentially expressed genes between two samples is described as follow:

Denote the number of unambiguous clean tags (which means reads in RNA-Seq) from gene A as *x*, given every gene's expression occupies only a small part of the library, *x* yields to the Poisson distribution:

(1) $$\begin{array}{r} p\left(x\right)=\frac{e^{-\lambda }\lambda^{x}}{x!}\left(\lambda \text{ is the real transcripts of the gene}\right) \end{array}$$

The total clean tag number of the sample 1 is N_1_, and total clean tag number of sample 2 is N_2_; gene A holds *x* tags in sample 1 and *y* tags in sample 2. The probability of gene A expressed equally between two samples can be calculated with:

(2) $$\begin{array}{r} 2\overset{i\;=\;y}{\underset{i\;=\;0}{\sum }}p\left(i|x\right)\text{or }2\times \left(1-\overset{i\;=\;y}{\underset{i\;=\;0}{\sum }}p\left(i|x\right)\right)\left(\text{if}\overset{i\;=\;y}{\underset{i\;=\;0}{\sum }}p\left(i|x\right)>0.5\right) \end{array}$$

(3) $$\begin{array}{r} p\left(y|x\right)={\left(\frac{N_{2}}{N_{1}}\right)}^{y}\frac{\left(x+y\right)!}{x!y!{\left(1+\frac{N_{2}}{N_{1}}\right)}^{\left(x+y+1\right)}} \end{array}$$

The full transcriptome sequencing data has been deposited in the NCBI SRA, with accession number SRP099178.

### Cell culture and cytotoxicity assay

The bacterial cytotoxicity was determined by the lactate dehydrogenase (LDH) release assay as previously described (Anderson et al., 2010; Li et al., 2016). HeLa cells (Li et al., 2016) were cultured in DMEM medium (Corning, USA) with 10% fetal bovine serum (FBS) supplemented with 1% penicillin/streptomycin (Gibco, USA) at 37°C in 5% CO_2_. 1.5 × 10^4^ HeLa cells were seeded into each well of a 96-well plate and cultured for 24 h. The medium was replaced with antibiotic and FBS free DMEM before *P. aeruginosa* infection. Overnight bacterial cultures were subcultured in fresh LB and grown to an OD_600_ of 1.0. Bacteria were washed once and resuspended in 1 × PBS. HeLa cells were infected with bacteria at a multiplicity of infection (MOI) of 50. The plate was centrifuged at 2,000 g for 10 min to synchronize the bacterial infection. After 3 h, LDH present in the supernatant was measured using the LDH cytotoxicity assay kit (Beyotime, China). Cells treated with 1% Triton X-100 were used as positive control of maximum release (100% percentage of cytotoxicity). The percentage of cytotoxicity was calculated following the manufacturer's instruction.

### Murine acute pneumonia model

All animal experiments complied with Nankai University and Chinese National Guidelines regarding the use of animals in research. The protocol was approved by the institutional animal care and use committee of the college of life sciences of Nankai University with permit number: NK-04-2012. Overnight bacterial cultures were subcultured in fresh LB at 37°C to an OD_600_ of 1.0. The bacterial cultures were centrifuged at 12 000 rpm for 1 min, and the pellets were resuspended in PBS. Each 6-week old female BALB/c mouse (Academy of Military Medical Sciences, Beijing, China) was anesthetized with 100 μL of 7.5% chloral hydrate by intraperitoneal injection. Then each mouse was intranasally inoculated with 1 × 10^7^ CFU bacteria. Bacterial colonization in the lung was determined as described previously (Sun et al., 2014). Briefly, 14 h post infection (hpi), mice were sacrificed by inhalation of CO_2_. Lungs were isolated and homogenized in 1% proteose peptone (Solarbio, Beijing, China). Bacterial loads were determined by plating serial dilutions and counting colonies. For the survival assay, the mice were intranasally inoculated with 2 × 10^7^ CFU bacteria and monitored for 5 days.

### RNA extraction from *In vivo* samples

Six week old female BALB/c mice were intranasally inoculated with 2 × 10^7^ CFU bacteria as described above. Mice were sacrificed by inhalation of CO_2_ at 6 hpi. Bronchoalveolar lavage fluid (BALF) was obtained by annulation of the trachea followed by twice instillations of 1 ml sterile PBS with 0.5 mM EDTA. 200 μl of the BALF was used for bacterial counting. The remaining BALF was centrifuged at 12,000 rpm for 2 min, and the pellets were resuspended in 200 μl TRIzol reagent (Thermo Fisher Scientific, USA). Total RNA was isolated using a Direct-zol RNA Miniprep kit (ZYMO research, USA).

### Electrophoretic mobility shift assay (EMSA)

EMSA was performed as previously described with minor modification (Sun et al., 2014). Briefly, DNA fragments corresponding to sequence upstream of *exsA* and *exsC* were synthesized. DNA fragments (200 ng) were incubated with 0 to 6 mM purified recombinant Fis protein at 25°C for 30 min in a 20-μl reaction [10 mM Tris-HCl, pH 7.5, 5 mM MgCl_2_, 5 mM KCl, 0.1% (v/v) NP-40 (Solarbio), and 1 mM dithiothreitol]. Samples were loaded onto an 8% native polyacrylamide gel in 0.5 × Tris-borate-EDTA (TBE) buffer (0.044 M Tris base, 0.044 M boric acid, 0.001 M EDTA, pH 8.0) that had been prerun for 1 h, electrophoresed on ice at 100 V for 1.5 h, followed by DNA staining in 0.5 × TBE containing 0.5 μg/ml ethidium bromide. Bands were visualized with a molecular imager ChemiDoc™ XRS+ (Bio-Rad).

### Western blotting

Overnight bacterial cultures were subcultured in fresh LB with or without 5 mM EGTA at 37°C for 3 h. The pellets from 1 ml cultures were then resuspended in 100 μl loading buffer (50 mM Tris-HCl, pH 6.8, 2% (w/v) SDS, 0.1% (w/v) BPB, 10% (v/v) Glycerol, 1% (v/v) 2-ME). Protein samples from equivalent amounts of protein were loaded onto a 15% SDS-PAGE gel. Proteins were separated by electrophoresis followed by transferring to a polyvinylidene difluoride (PVDF) membrane (Millipore, USA). The target proteins were hybridized with a rabbit monoclonal anti-His antibody (CST, USA) or a mouse monoclonal anti-Flag antibody (Sigma, USA). Signals were detected with an Immobilon™ Western kit (Millipore).

### Transcriptional reporter assay

Overnight bacterial cultures were subcultured in fresh LB with or without 5 mM EGTA at 37°C with shaking, and samples were harvested when OD_600_ of the cultures reached 1.0. The β-galactosidase activity was measured with substrate ortho-nitrophenyl-galactopyranoside (ONPG) (BBI life sciences) as previously described (Ha et al., 2004).

### Statistical analysis

The statistical analyses were performed with the Prism software (Graphpad Software). The real time PCR and β-galactosidase assay results were analyzed by the Student's *t*-test (two-tailed). Bacterial colonization results were analyzed with the Mann-Whitney test. Survival data were analyzed with the Log-rank (Mantel-Cox) test.

## Results

### Fis is essential for the virulence of *P. aeruginosa* in a mouse acute pneumonia model

**Figure 1:**
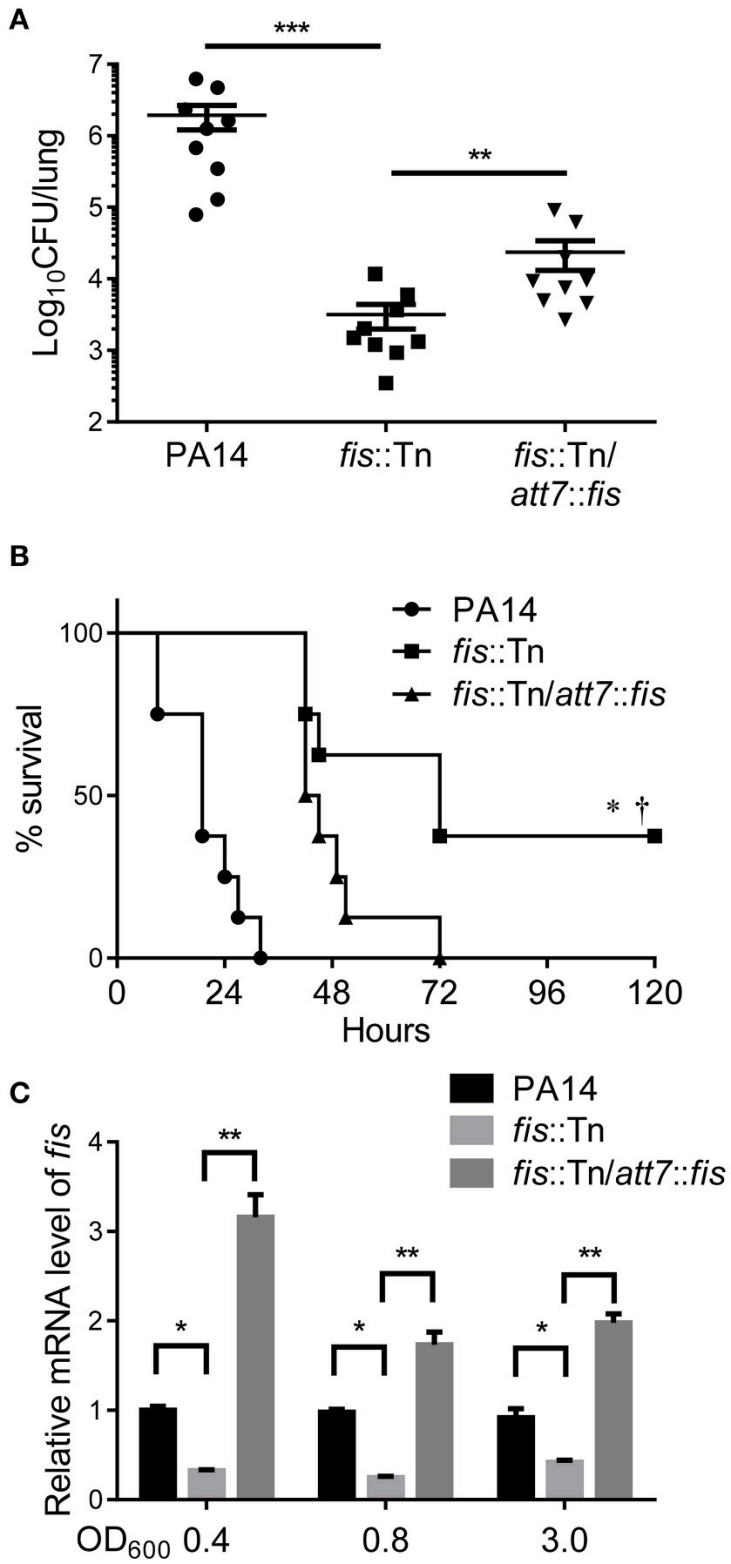
**Fis is essential for the bacterial virulence ***in vivo***. (A)** Mice were inoculated intranasally with 1 × 10^7^ CFU bacteria of indicated strains. 14 hpi, mice were sacrificed and lungs were isolated and homogenized. Bacterial loads were determined by serial dilution and plating. The central bar indicates the mean, and error bars indicate standard error of the mean. ^***^p < 0.001; ^**^p < 0.01 by the Mann-Whitney test. **(B)** Mice were inoculated intranasally with 2 × 10^7^ CFU bacteria of indicated strains. The mice were monitored for 5 days after the infection. ^*^p < 0.001, compared to wild type PA14 by log-rank (Mantel-Cox) test; ^†^*p* < 0.05, compared to the complemented strain (*fis*::Tn/*att7*::*fis*) by log-rank (Mantel-Cox) test. **(C)** Total RNA was isolated from bacterial culture at indicated optical densities (OD_600_). cDNA was synthesized with *fis* and *rpsL* specific primers. Relative mRNA levels of *fis* were determined by quantitative real-time PCR. The 30S ribosomal protein coding gene *rpsL* was used as an internal control. Data represents the mean ± standard deviation from three samples. ^*^p < 0.05; ^**^p < 0.01 by Student's *t*-test.

To examine the role of Fis in the virulence of *P. aeruginosa*, we infected mice with wild type PA14 or a *fis*::Tn mutant from the nonredundant library of PA14 transposon mutants (Liberati et al., 2006) in an acute pneumonia model. 14 h post infection, lungs were isolated and the bacterial numbers were determined by serial dilution and plating. Compared to the wild type strain, the number of the *fis*::Tn mutant was significantly lower (Figure 1A). Complementation with a *fis* gene driven by its native promoter partially restored the bacterial load (Figure 1A). To confirm the role of Fis in virulence, we monitored the survival rate in the acute pneumonia model. Infection with wild type PA14 or the complemented strain caused death in all the infected mice, whereas infection with the *fis*::Tn resulted in 40% survival rate (Figure 1B).

In the PA14 *fis*::Tn mutant, the Tn was inserted right before the stop codon of the *fis* gene (Liberati et al., 2006). To examine the effect of Tn insertion on the expression of Fis, we determined the mRNA level of Fis by real time PCR. Compared to wild type PA14, the mRNA level of Fis was lower in the *fis*::Tn mutant, but higher in the complemented strain in bacteria at early-, mid-log and stationary growth phases (Figure 1C). Thus, the excessive expression of Fis in the complemented strain might affect bacterial virulence, resulting in partial restoration of bacterial number in lung and slower killing of infected mice (Figures 1A,B).

The *fis* gene is in the same operon with PA4852. Thus, the expression of PA4852 might be affected by the Tn insertion. A real time PCR assay revealed similar mRNA levels of PA4852 in wild type PA14 and the *fis*::Tn mutant (Figure S1), suggesting that the Tn insertion did not affect the expression of PA4852. It has been demonstrated that the translation of Fis is repressed by the small RNA RgsA (Lu et al., 2016). The RgsA level in the *fis*::Tn mutant was similar as that in the wild type PA14 (Figure S2), suggesting that the RgsA mediated regulation on Fis might be normal in the *fis*::Tn mutant. In combination, these results suggest that the lower expression of Fis might result in attenuation of the virulence of PA14. Since *fis* has been indicated as an essential gene in *P. aeruginosa*, we used the *fis*::Tn mutant in our further studies (Liberati et al., 2006; Jakovleva et al., 2012).

### Fis is required for expression of the T3SS and for cytotoxicity

**Figure 2:**
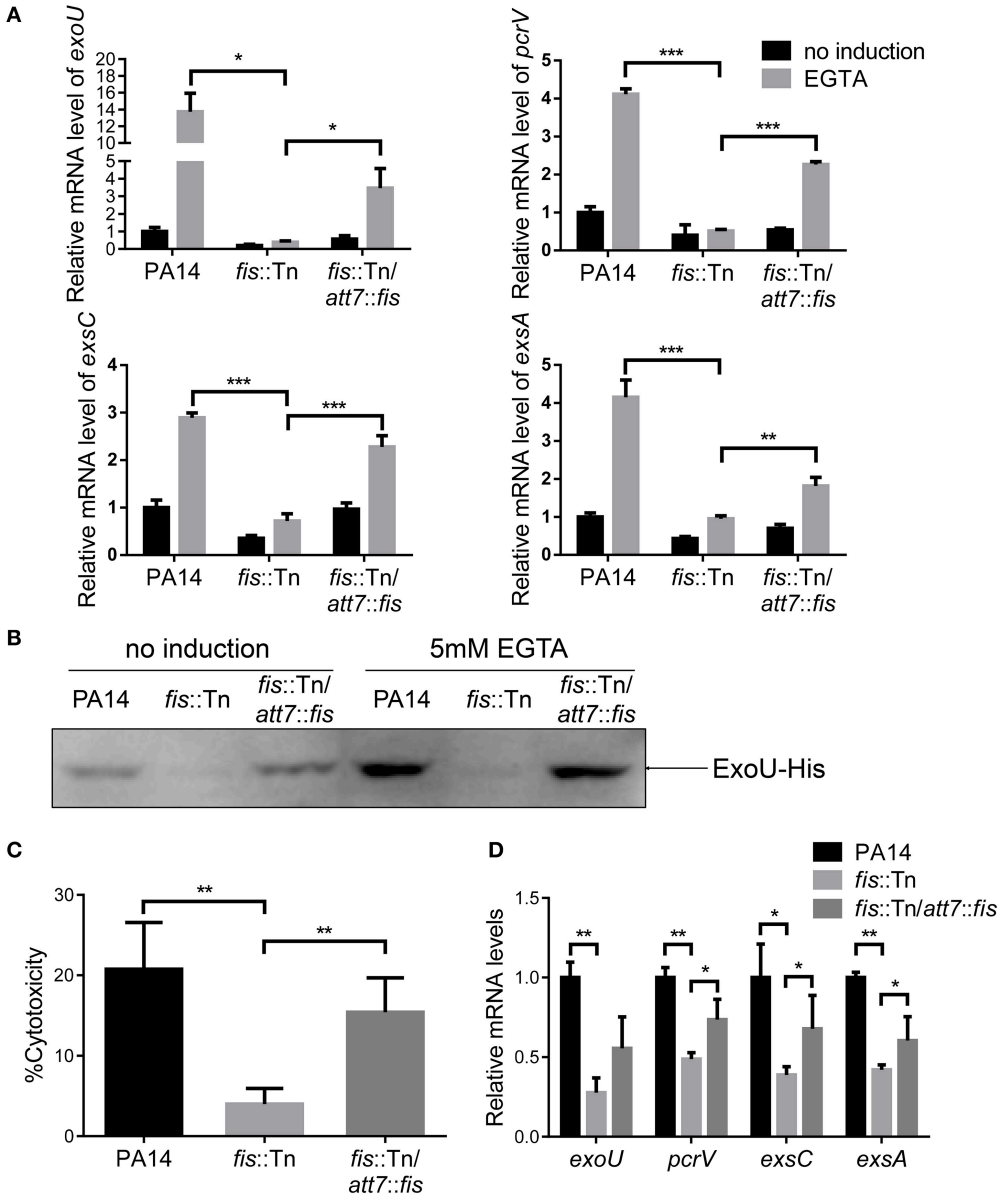
**Fis is required for T3SS gene expression and bacterial cytotoxicity. (A)** Relative mRNA levels of T3SS genes *exoU, pcrV, exsC, exsA*. Total RNA was isolated from bacteria grown with or without 5 mM EGTA and relative mRNA levels of these genes were determined by quantitative real-time PCR. Data represents the mean ± standard deviation from three samples. ^*^p < 0.05; ^**^p < 0.01; ^***^p < 0.001 by Student's *t*-test. **(B)** PA14, *fis*::Tn mutant and *fis*::Tn/*att7*::*fis* carrying an *exoU*-His driven by its native promoter (P_*exoU*_-*exoU*-His) were grown at 37°C with or without 5 mM EGTA for 3 h. Proteins samples from equal amounts of protein were separated by SDS-PAGE and the ExoU-His levels were determined by western blotting analysis using an anti-His antibody. **(C)** Bacterial cytotoxicity on HeLa cells. HeLa cells were infected with indicated strains at a MOI of 50 for 3 h. The bacterial cytotoxicity was determined by the LDH release assay. Error bars indicate standard deviations of triplicate assays. ^**^p < 0.01 by Student's *t*-test. **(D)** Relative mRNA levels of T3SS genes during lung infection. Mice were infected intranasally with indicated strains. 6 hpi, bacteria from BALF were collected, followed by RNA isolation. Relative mRNA levels of *exoU, pcrV, exsC*, and *exsA* were determined by quantitative real-time PCR. Data represents the mean ± standard deviation from three independent experiments. ^*^p < 0.05; ^**^p < 0.01 by Student's *t*-test.

To understand how Fis affects bacterial virulence, we compared transcriptome profiles between the *fis*::Tn mutant and wild type PA14. Interestingly, the T3SS genes were down regulated in the *fis*::Tn mutant (Table 1). To confirm the expression levels of T3SS genes, bacteria were cultured under T3SS non-inducing and inducing conditions. Real time PCR assay revealed lower mRNA levels of *exoU, pcrV, exsC*, and *exsA* in the *fis*::Tn mutant, which were partially restored in the complemented strain (Figure 2A). By utilizing a transcriptional fusion of the *exsC* promoter and a *lacZ* gene (P_*exsC*_-*lacZ*), we found the *exsC* promoter activity was lower in the *fis*::Tn mutant (Figure S3). To confirm the expression level of T3SS genes, a His-tagged *exoU* (ExoU-His) driven by its native promoter was transferred into the bacteria. As shown in Figure 2B, the ExoU-His level was reduced in the *fis*::Tn mutant under T3SS inducing condition. Since T3SS plays a major role in bacterial cytotoxicity (Yahr and Wolfgang, 2006; Tan et al., 2016), we infected HeLa cells with PA14, the *fis*::Tn mutant and the complemented strain. Consistent with the mRNA levels of T3SS genes, the *fis*::Tn mutant displayed reduced cytotoxicity to HeLa cells (Figure 2C). We then examined the expression levels of T3SS genes in the murine acute pneumonia model. Bacteria were isolated from BALF at 6 hpi. mRNA levels of *exoU, pcrV, exsC* and *exsA* were lower in the *fis*::Tn mutant, which was partly restored in the complemented strain (Figure 2D).

**Table 1:** **mRNA levels of T3SS genes in the ***fis***::Tn mutant compared to wild type PA14**.

**Locus tag (PA14)**	**Locus tag (PAO1)**	**Gene name**	**Product description**	**Fold changes (*fis*::Tn/PA14)**	***P*–value**
PA14_RS00230	PA0044	*exoT*	Exoenzyme T	0.63	1.71E–57
PA14_RS14785	PA2191	*exoY*	Adenylate cyclase ExoY	0.61	1.65E–06
PA14_RS17140	PA1725	*pscL*	Type III export protein PscL	0.36	1.23E–14
PA14_RS17145	PA1724	*pscK*	Type III export protein PscK	0.45	1.21E–08
PA14_RS17150	PA1723	*pscJ*	Type III export protein PscJ	0.36	4.23E–33
PA14_RS17155	PA1722	*pscI*	Type III export protein PscI	0.36	2.14E–19
PA14_RS17160	PA1721	*pscH*	Type III export protein PscH	0.34	3.93E–22
PA14_RS17165	PA1720	*pscG*	Type III export protein PscG	0.37	1.30E–28
PA14_RS17170	PA1719	*pscF*	Type III export protein PscF	0.31	1.50E–20
PA14_RS17175	PA1718	*pscE*	Type III export protein PscE	0.30	8.29E–14
PA14_RS17180	PA1717	*pscD*	Type III export protein PscD	0.32	3.45E–54
PA14_RS17185	PA1716	*pscC*	Type III secretion outer membrane protein PscC precursor	0.41	8.84E–51
PA14_RS17190	PA1715	*pscB*	Type III export apparatus protein	0.48	4.42E–12
PA14_RS17195	PA1714	*exsD*	exsD	0.50	1.18E–55
PA14_RS17200	PA1713	*exsA*	Transcriptional regulator ExsA	0.48	6.83E–18
PA14_RS17205	PA1712	*exsB*	Exoenzyme S synthesis protein B	0.46	1.72E–25
PA14_RS17210	PA1711	*exsE*	exsE	0.55	2.65E–17
PA14_RS17215	PA1710	*exsC*	Exoenzyme S synthesis protein C	0.56	4.77E–38
PA14_RS17220	PA1709	*popD*	Translocator outer membrane protein PopD precursor	0.44	1.98E–153
PA14_RS17225	PA1708	*popB*	Translocator protein PopB	0.41	4.86E–264
PA14_RS17230	PA1707	*pcrH*	Regulatory protein PcrH	0.51	3.10E–26
PA14_RS17235	PA1706	*pcrV*	Type III secretion protein PcrV	0.58	1.63E–23
PA14_RS17240	PA1705	*pcrG*	Regulator in type III secretion	0.40	3.69E–09
PA14_RS17245	PA1704	*pcrR*	Transcriptional regulator protein PcrR	0.50	7.05E–04
PA14_RS17250	PA1703	*pcrD*	Type III secretory apparatus protein PcrD	0.47	1.23E–24
PA14_RS17255	PA1702	*pcr4*	pcr4	0.72	2.30E–01
PA14_RS17260	PA1701	*pcr3*	pcr3	0.45	5.09E–04
PA14_RS17265	PA1700	*pcr2*	pcr2	0.65	3.65E–02
PA14_RS17270	PA1699	*pcr1*	pcr1	0.59	4.82E–03
PA14_RS17275	PA1698	*popN*	Type III secretion outer membrane protein PopN precursor	0.62	9.97E–07
PA14_RS17280	PA1697	*pscN*	ATP synthase in type III secretion system	0.42	1.19E–19
PA14_RS17285	PA1696	*pscO*	Translocation protein in type III secretion	0.24	4.09E–09
PA14_RS17290	PA1695	*pscP*	Translocation protein in type III secretion	0.27	3.01E–06
PA14_RS17295	PA1694	*pscQ*	Translocation protein in type III secretion	0.36	2.37E–16
PA14_RS17300	PA1693	*pscR*	Translocation protein in type III secretion	0.43	2.80E–06
PA14_RS17305	PA1692	*pscS*	Probable translocation protein in type III secretion	0.36	1.66E–05
PA14_RS17310	PA1691	*pscT*	Translocation protein in type III secretion	0.38	4.66E–07
PA14_RS17315	PA1690	*pscU*	Translocator protein PopB	0.65	1.10E–03
PA14_RS20955	−	*spcU*	spcU	0.61	2.83E–14
PA14_RS20960	−	*exoU*	Exoenzyme U	0.51	2.19E–141

**Figure 3:**
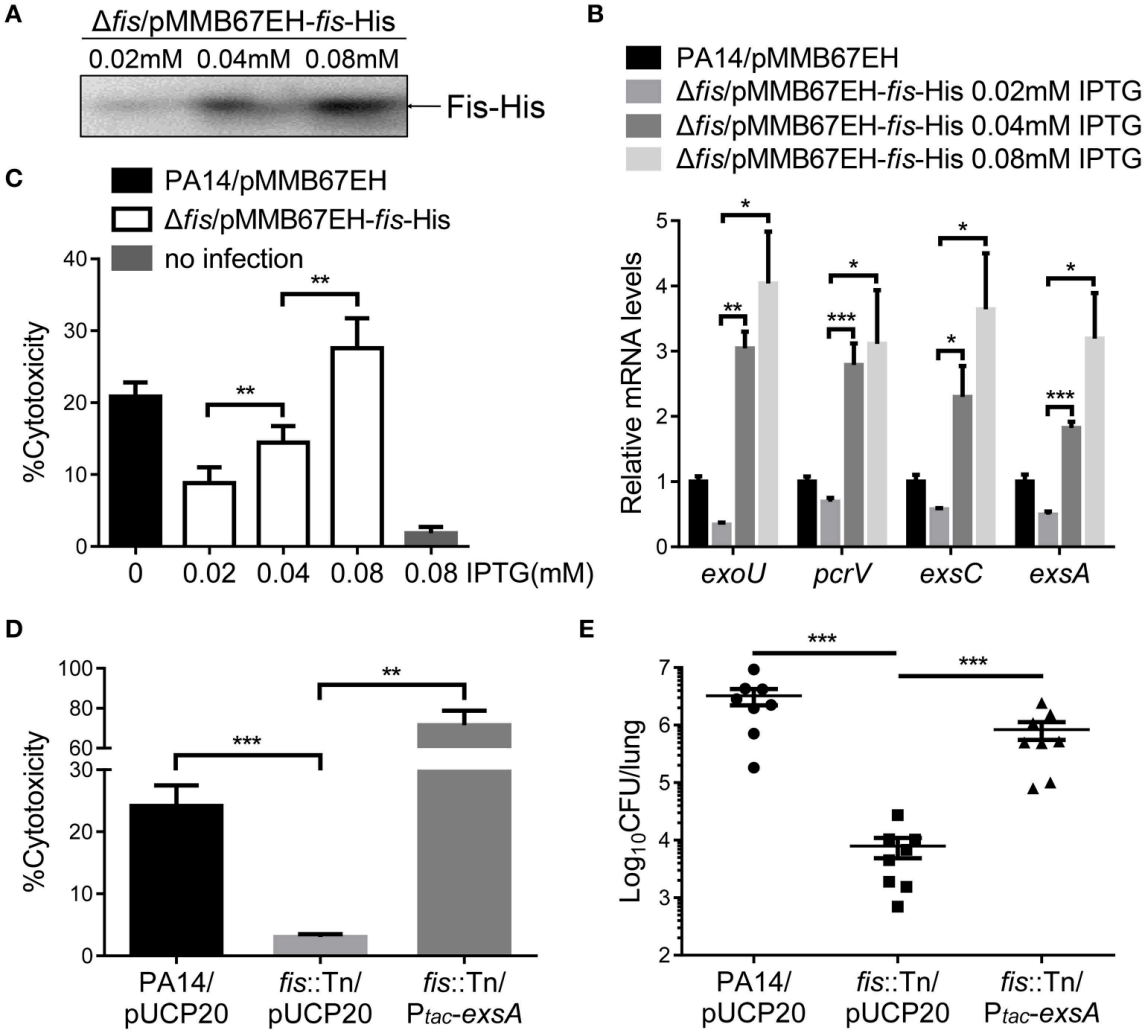
**Fis is required for the activation of the T3SS. (A)** The strain Δ*fis*/pMMB67EH-*fis*-His was grown at 37°C with indicated concentrations of IPTG to an OD_600_ of 1.0. Proteins samples from equal amounts of protein were separated by SDS-PAGE and the Fis-His levels were determined by western blotting analysis using an anti-His antibody. **(B)** Relative mRNA levels of T3SS genes. Total RNA of indicated strains was isolated from bacteria grown with indicated concentrations of IPTG and mRNA levels of T3SS genes were determined by quantitative real time PCR. Data represents the mean ± standard deviation from three samples. ^*^p < 0.05; ^**^p < 0.01; ^***^p < 0.001 by Student's *t*-test. **(C)** Bacterial cytotoxicity on HeLa cells. HeLa cells were infected with indicated strains with indicated concentration of IPTG at a MOI of 50 for 3 h. The bacterial cytotoxicity was determined by the LDH release assay. Error bars indicate standard deviations of triplicate assays. ^**^p < 0.01, by Student's *t*-test. **(D)** HeLa cells were infected with indicated strains at a MOI of 50 for 3 h. The bacterial cytotoxicity was determined by the LDH release assay. Error bars indicate standard deviations of triplicate assays. ^**^p < 0.01; ^***^p < 0.001 by Student's *t*-test. **(E)** Mice were inoculated intranasally with 1 × 10^7^ CFU bacteria of indicated strains. 14 hpi, mice were sacrificed and lungs were isolated and homogenized. Bacterial loads were determined by serial dilution and plating. The central bar indicates the mean, and error bars indicate standard error of the mean. ^***^p < 0.001 by the Mann-Whitney test.

To further confirm the role of Fis in the regulation of T3SS, we cloned a C-terminus His-tagged *fis* gene (*fis*-His) into pMMB67EH (Fürste et al., 1986), where the expression of *fis*-His is driven by an inducible *tac* promoter. In the presence of this plasmid, we deleted the chromosomal *fis* gene, resulting in the strain Δ*fis*/pMMB67EH-*fis*-His. In the absence of IPTG, the strain could not grow up in LB, confirming the essential role of Fis in *P. aeruginosa*. For an unknown reason, EGTA significantly repressed the growth of the strain Δ*fis*/pMMB67EH-*fis*-His in the presence of various concentrations of IPTG. Thus, we only examined the expression of T3SS genes in LB medium. In the presence of 0.02 mM IPTG, the RNA levels of T3SS genes were lower in the Δ*fis*/pMMB67EH-*fis*-His strain. Increasing amount of IPTG resulted in higher amount of Fis-His protein and up regulation of the T3SS genes (Figures 3A,B). Next, we examined the bacterial cytotoxicity with IPTG in the tissue culture medium. At low concentration of IPTG, the bacteria displayed minimal cytotoxicity, whereas the presence of IPTG increased the bacterial cytotoxicity (Figure 3C). In combination, these results suggest that Fis is required for the activation of the T3SS.

In LB broth, the growth rate of the *fis*::Tn mutant was similar as that of the wild type PA14 (Figure S4). However, it has been reported that the *fis*::Tn mutant grows more slowly in BM2 swarming medium (Yeung et al., 2009), indicating a role of Fis in bacterial growth under certain conditions. Thus, the lower bacterial loads in the lungs from the *fis*::Tn mutant infected mice might be due to slower bacteria growth rate. To verify the role of T3SS in the reduced colonization of the *fis*::Tn mutant, we overexpressed *exsA* in the *fis*::Tn mutant and examined bacterial cytotoxicity and colonization of lungs. As shown in Figure S5, overexpression of *exsA* did not affect the growth of the *fis*::Tn mutant. Overexpression of *exsA* in the *fis*::Tn mutant restored bacterial cytotoxicity (Figure 3D) and colonization *in vivo* (Figure 3E). Therefore, the defective T3SS indeed attributed to the attenuated virulence of the *fis*::Tn mutant.

### Fis directly interacts with *exsA* promoter region

**Figure 4:**
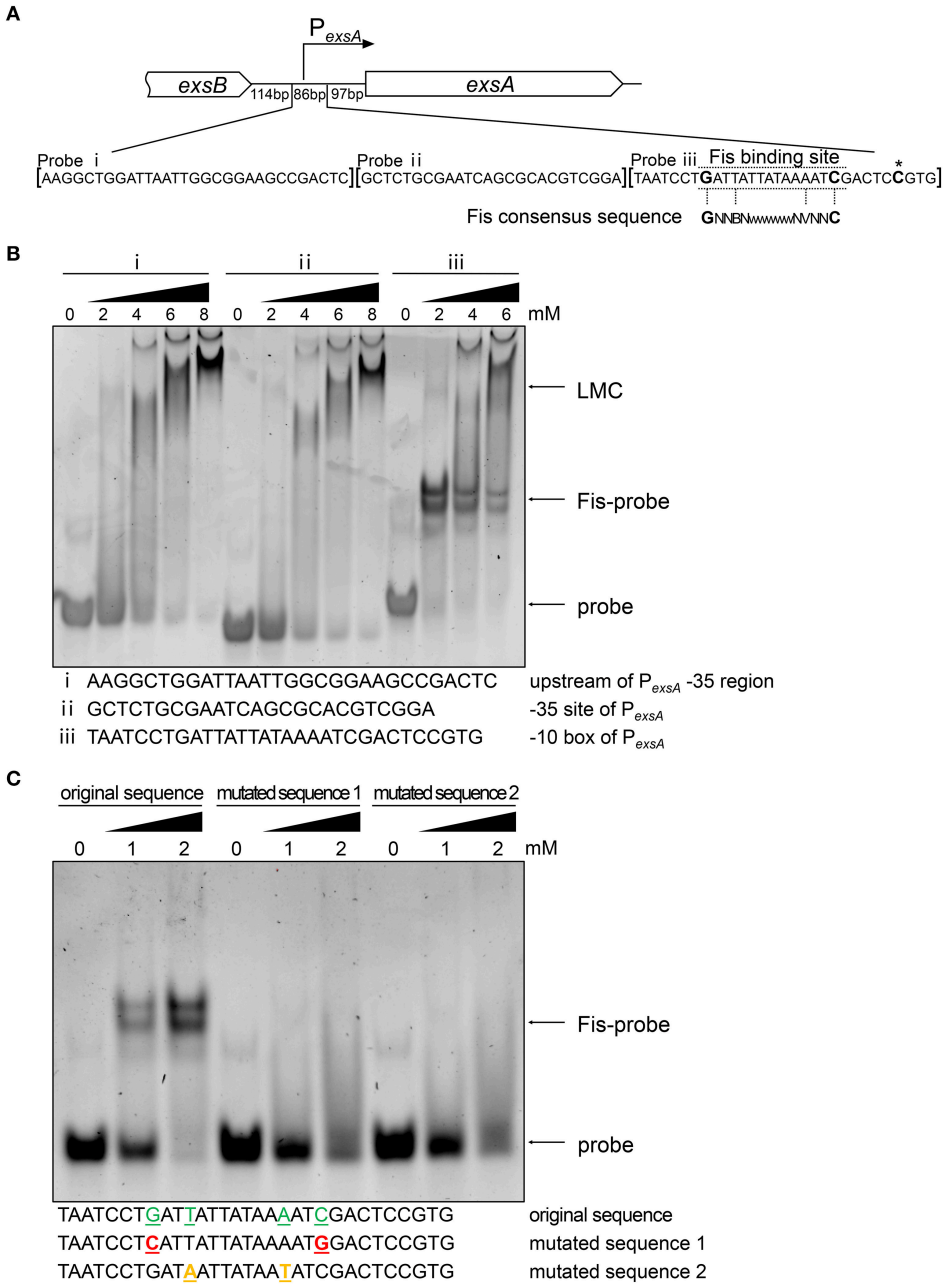
**Fis directly interacts with ***exsA*** promoter region. (A)** Diagram of the *exsA* promoter region. P_*exsA*_ is indicated by an arrow. Sequences of the probes used in EMSA and the consensus Fis binding sequence are shown. The transcription start site of *exsA* is indicated by an asterisk. Fragment i, ii, and iii represent DNA probes used in the EMSA. **(B)** Fis was incubated with probe i, ii, or iii for 30 min at 25°C. Arrows indicate the positions of unbound probes or the Fis-probe complex and LMC. **(C)** Binding of the Fis to the DNA fragment containing P_*exsA*_ -10 box original and mutated sequences. Colored underlined letters represent mutated nucleotides. Arrows indicate positions of the Fis-probe complex and unbound probes, respectively.

T3SS genes are directly regulated by ExsA (Hauser, 2009; Diaz et al., 2011) and we have found that the mRNA level of *exsA* was lower in the *fis*::Tn mutant (Figures 2A,D). Since Fis functions as a transcriptional factor (TF) (Muskhelishvili et al., 1995; Schneider et al., 2001; Aiyar et al., 2002; Cho et al., 2008; Kahramanoglou et al., 2011; Prigent-Combaret et al., 2012), we suspected Fis might directly affect the transcription of *exsA*. By carefully searching for the consensus Fis binding motif (Cho et al., 2008; Shao et al., 2008; Kahramanoglou et al., 2011; Hancock et al., 2016), we identified a possible Fis binding site at the -10 box of the *exsA* promoter (Figure 4A). An EMSA assay demonstrated an interaction between Fis and this fragment (Figure 4B). As a control, the fragments up stream of the binding site could not bind to Fis at the concentration of 2 mM. Of note, at higher concentrations of Fis, DNA retardation was observed with all the tested probes (Figure 4B), which might be attributed to the nonspecific coating of DNA by Fis, resulting in the formation of Fis-DNA filament referred to as a “low mobility complex (LMC)” (Skoko et al., 2006). To further verify the specific binding between Fis and the -10 box of the P_*exsA*_, we mutated the conserved nucleotides of the Fis binding site based on the study in *E. coli*. The replaced nucleotides were highlighted in Figure 4C. Indeed, alternation of the highly conserved nucleotides abolished the interaction between Fis and the fragment (Figure 4C). Since ExsC positively regulates T3SS (Hauser, 2009; Diaz et al., 2011), we also examined whether Fis specifically binds to the *exsC* promoter region. However, no specific binding between Fis and the test fragments were observed (Figure S6). These results indicated that Fis directly binds to the -10 box of P_*exsA*_.

### Role of Fis in the P*_*exsA*_* activity

**Figure 5:**
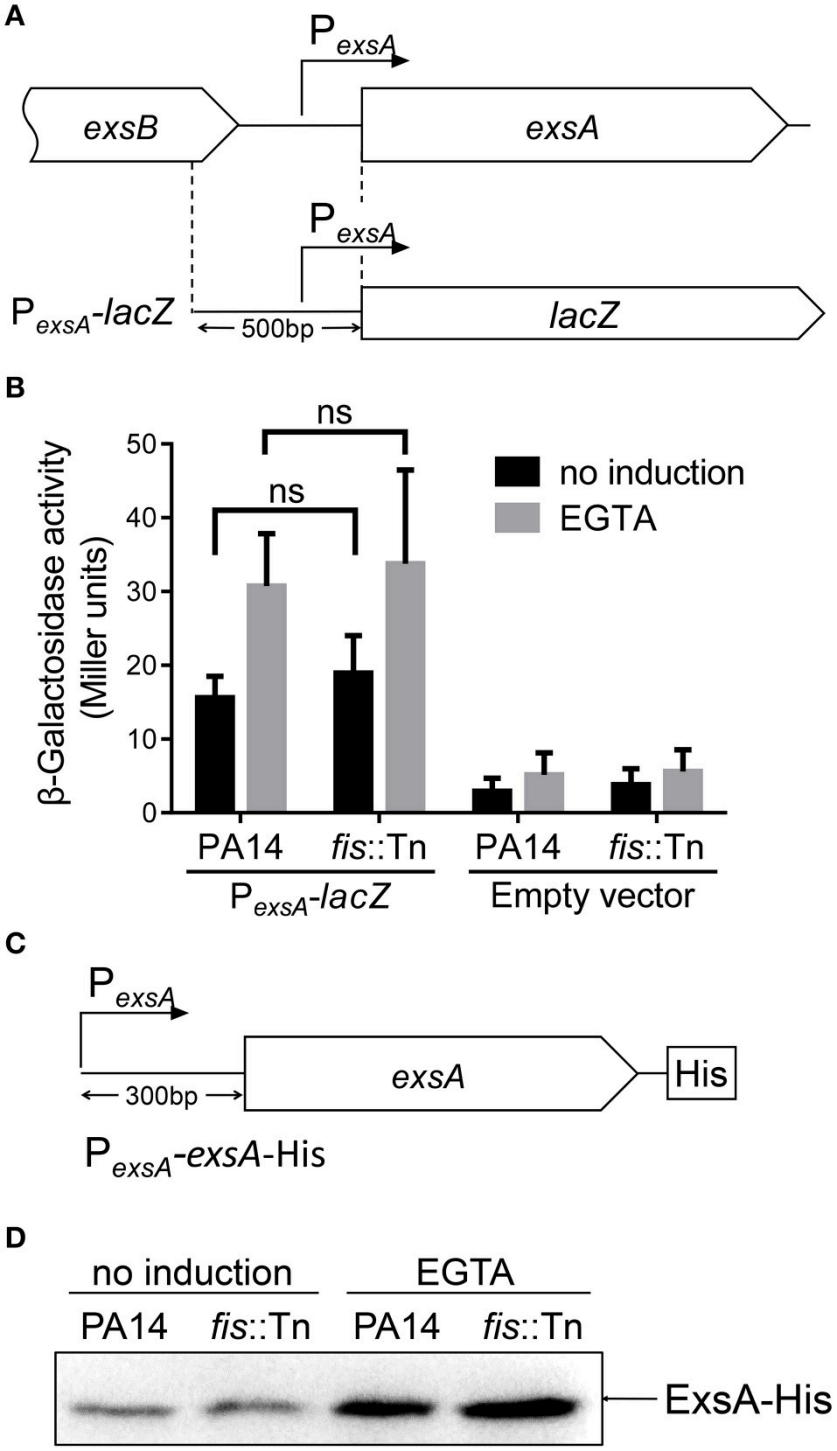
**Role of Fis in the regulation of P_***exsA***_. (A)** Diagram of the P_*exsA*_*-lacZ* transcriptional fusion. **(B)** PA14 and the *fis*::Tn mutant carrying the P_*exsA*_*-lacZ* fusion reporter or empty vector (promoterless *lacZ*) were grown at 37°C with or without 5 mM EGTA for 3 h. The values (Miller units) are the means of three experiments. ns, not significant by Student's *t*-test. **(C)** Diagram of the P_*exsA*_*-exsA-*His construct. The *exsA* open reading frame with its upstream 300 bp region was fused with a 6 × His tag at the C-terminus. **(D)** PA14 and the *fis*::Tn mutant carrying an *exsA*-His driven by its native promoter (P_*exsA*_-*exsA*-His) were grown at 37°C with or without 5 mM EGTA for 3 h. Proteins samples from equal amounts of protein were separated by SDS-PAGE. The ExsA-His levels were determined by western blotting analysis with an anti-His antibody.

The specific binding between Fis and the P_*exsA*_ region raised a possibility that Fis affects the activity of P_*exsA*_. To test this possibility, we constructed a P_*exsA*_-*lacZ* transcriptional fusion (Figure 5A). The β-galactosidase levels were similar between wild type PA14 and the *fis*::Tn mutant under either T3SS non-inducing or inducing condition (Figure 5B). To confirm this observation, we constructed a C-terminus His-tagged ExsA driven by the P_*exsA*_ (Figure 5C). Consistent with the P_*exsA*_-*lacZ* reporter result, similar ExsA-His protein levels were observed in wild type PA14 and the *fis*::Tn mutant (Figure 5D). These results suggest that Fis might not directly affect the P_*exsA*_ activity.

### *exsA* transcription relies mainly on the transcription initiated from P*_*exsC*_*

**Figure 6:**
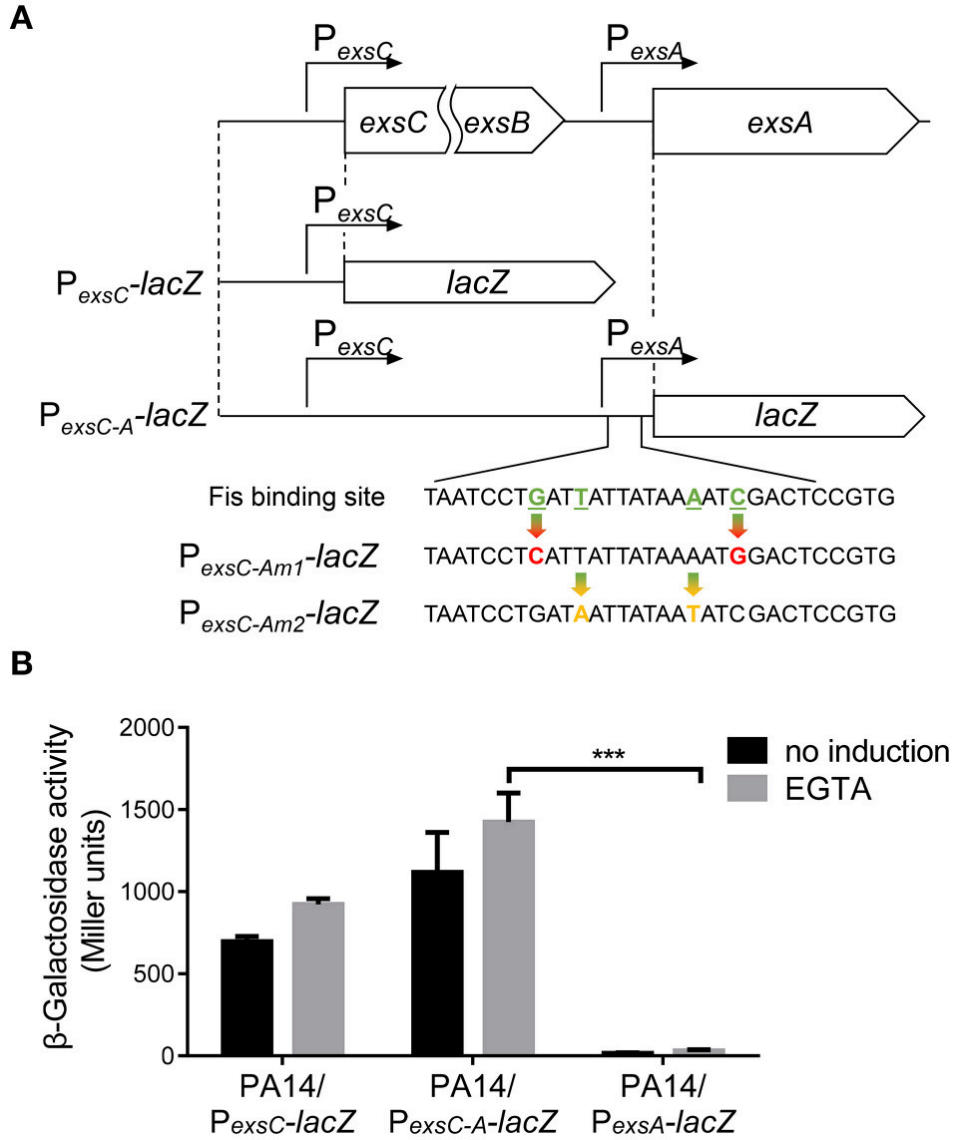
**Transcription driven by the ***exsC*** or ***exsA*** promoter. (A)** Diagram of the P_*exsC*_*-lacZ* and P_*exsC-A*_*-lacZ* transcriptional fusions. Point mutations of Fis binding site in P_*exsC-Am*1_*-lacZ* and P_*exsC-Am*2_*-lacZ* transcriptional fusions are indicated by arrows. **(B)** PA14 carrying P_*exsC*_*-lacZ*, P_*exsC-A*_*-lacZ* or P_*exsA*_*-lacZ* were grown at 37°C with or without 5 mM EGTA and assayed for β-galactosidase activities. The reported values (Miller units) are the means of results of at least three independent experiments. ^***^p < 0.001 by Student's *t*-test.

**Figure 7:**
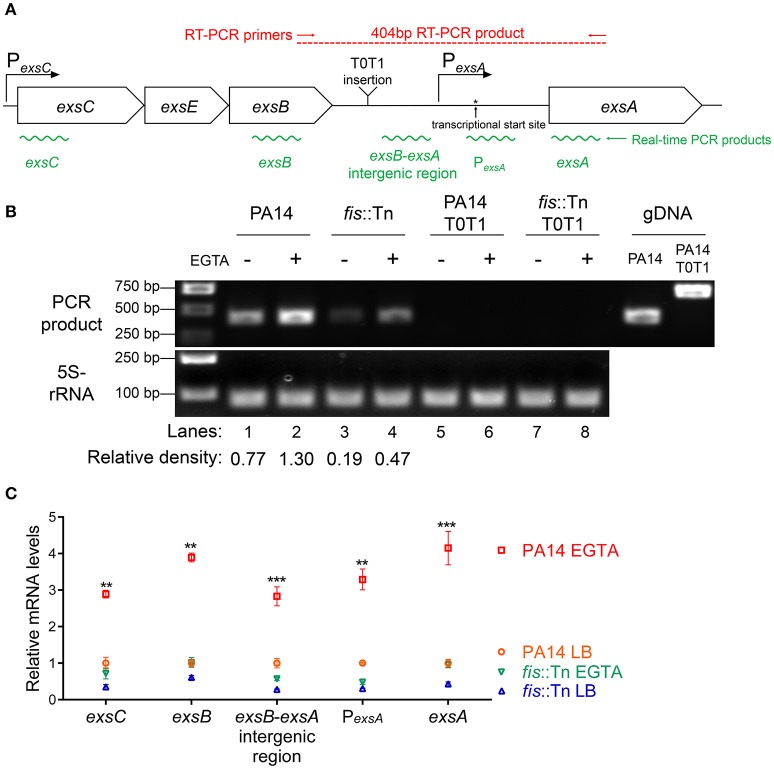
**Transcription of ***exsC***, ***exsE***, ***exsB*** and ***exsA***. (A)** Diagram of the *exsCEBA* operons. P_*exsC*_ and P_*exsA*_ are indicated by arrows. Red arrows indicate the directions and locations of the primers for RT-PCR. Insertion site of the transcriptional terminator T0T1 is indicated. Green wavy lines indicate positions of real time PCR products. **(B)** Total RNA was isolated from indicated strains grown with or without 5 mM EGTA for 3 h. cDNA was synthesized and used as templates in PCR. The 5S rRNA was used as an internal control. The density of each band in lanes 1–4 was determined with ImageJ (ImageJ software k1.45). The relative density was calculated by dividing the density of each *exsB*-*exsA* RT-PCR product by that of the corresponding 5S rRNA RT-PCR product **(C)** Relative mRNA levels of five regions within the *exsCEBA* operon. PA14 and *fis*::Tn mutant were grown in the presence or absence of 5 mM EGTA. The value of each tested fragment represents the RNA level relative to that in wild type PA14 grown in LB medium. Data represents the mean ± standard deviation from three independent experiments. ^**^p < 0.01, ^***^p < 0.001, compared to wild type PA14 grown in LB medium by Student's *t*-test.

Previously studies demonstrated that the P_*exsA*_ transcriptional activity is much weaker than P_*exsC*_ (Yahr and Frank, 1994; Marsden et al., 2016). We observed the same results under both T3SS non-inducing and inducing conditions (Figure 6B). It has been speculated that transcription of *exsA* might be driven by the *exsC* promoter under T3SS inducing condition (Hauser, 2009; Diaz et al., 2011). If this is the case, Fis might be involved in the regulation of *exsA* transcription initiated from the *exsC* promoter. To test this hypothesis, we firstly examined whether *exsCEBA* are in one transcript. We constructed a P_*exsC-A*_-*lacZ* transcriptional fusion, where the *lacZ* gene was cloned downstream of a fragment ranging from P_*exsC*_ to *exsA* coding region (Figure 6A). In wild type PA14, the β-galactosidase levels were much higher than that driven by P_*exsA*_ (P_*exsA*_-*lacZ*), indicating a continuous transcription from P_*exsC*_ to *exsA* coding region (Figure 6B). We then designed a pair of primers annealing to the coding regions of *exsB* and *exsA*, thus the PCR product spans the intergenic region between *exsB* and *exsA* (Figure 7A). Total RNA was isolated from PA14 grown under T3SS non-inducing and inducing conditions, followed by RT-PCR. PCR products were observed at both conditions, with higher amount under T3SS inducing condition (Figure 7B), indicating a transcript from *exsB* to *exsA*. To further confirm the continuous transcription from *exsB* to *exsA*, we performed real time PCR with primers annealing to the coding regions of *exsC, exsB, exsA* as well as the *exsB*-*exsA* intergenic region (Figure 7A). The mRNA levels of all the tested genes and the RNA level of the *exsB*-*exsA* intergenic region were induced by EGTA in wild type PA14 (Figure 7C). These results suggest that *exsA* and *exsB* are in one transcript and the transcription level responses to the T3SS inducing signal.

**Figure 8:**
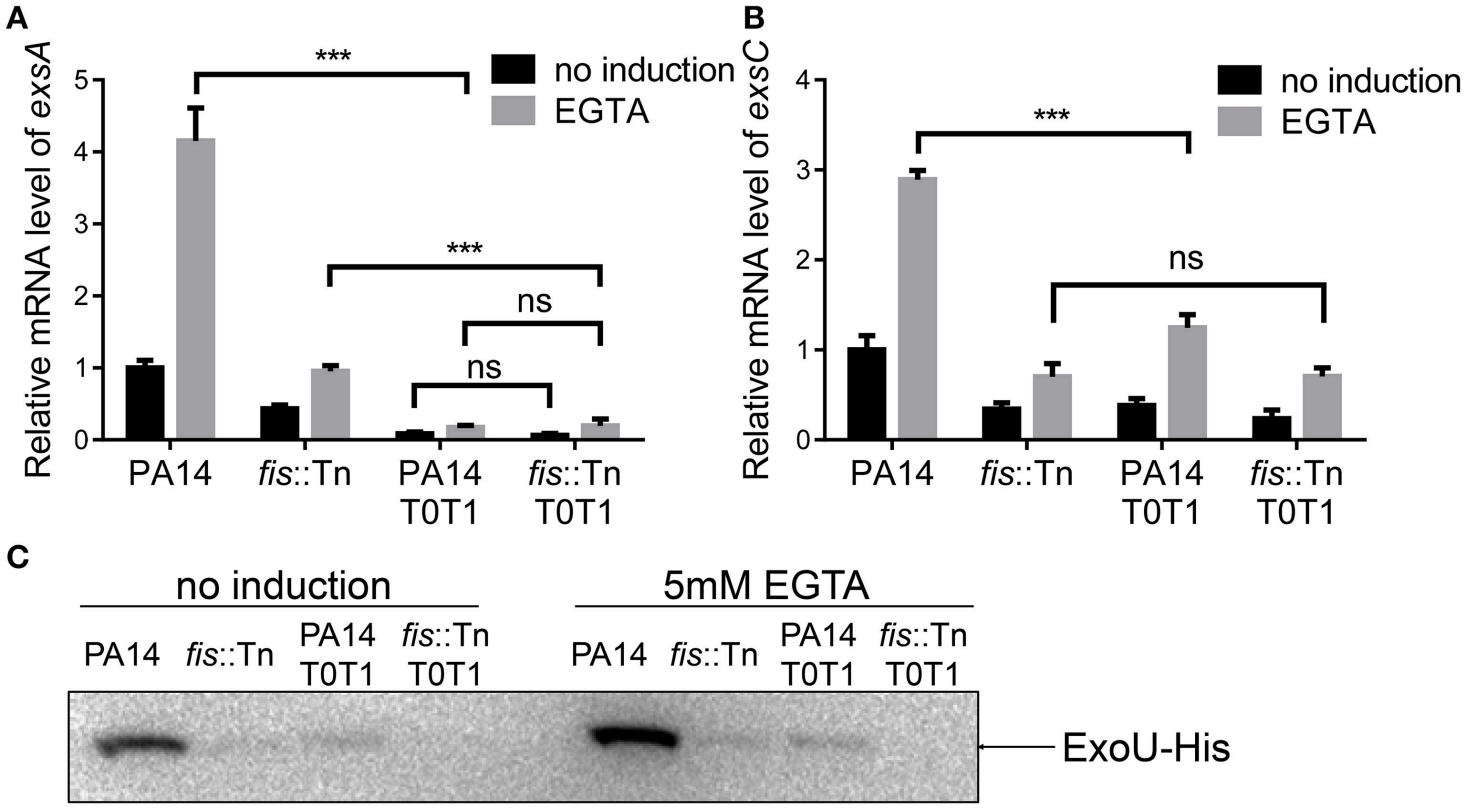
*****exsA*** transcription relies mainly on P_***exsC***_. (A)** Relative mRNA levels of *exsA*. Total RNA of indicated strains was isolated from bacteria grown with or without 5 mM EGTA and mRNA levels of *exsA* were determined by quantitative real time PCR. Data represents the mean ± standard deviation from three samples. ^***^p < 0.001 by Student's *t*-test. **(B)** Relative mRNA levels of *exsC*. Total RNA of indicated strains was isolated from bacteria grown with or without 5 mM EGTA and mRNA levels of *exsC* were determined by quantitative real time PCR. Data represents the mean ± standard deviation from three samples. ^***^p < 0.001 by Student's *t*-test. **(C)** PA14, *fis*::Tn mutant, PA14 T0T1 or *fis*::Tn T0T1 carrying an *exoU*-His driven by its native promoter (P_*exoU*_-*exoU*-His) were grown at 37°C with or without 5 mM EGTA for 3 h. Proteins samples from equal amounts of protein were separated by SDS-PAGE. ExoU-His levels were determined by western blotting analysis using an anti-His antibody.

If the P_*exsC*_ driven transcription of *exsA* is required for the activation of the T3SS, interception of the transcription between *exsB* and *exsA* coding regions should diminish the expression of *exsA* and consequently other T3SS genes. To test this hypothesis, we inserted two tandem transcription terminators (T0T1) up stream of the *exsA* promoter (Figure 7A). RT-PCR confirmed the break between *exsB* and *exsA* transcript (Figure 7B, lanes 5–8). Indeed, the *exsA* mRNA levels were significantly reduced by the insertion of T0T1 under both T3SS inducing and non-inducing conditions (Figure 8A). Consequently, the levels of *exsC* mRNA and the ExoU protein were reduced by the T0T1 insertion (Figures 8B,C). Therefore, the P_*exsC*_ driven transcription of *exsA* plays an essential role in the expression of T3SS genes.

### Fis is required for the transcription of *exsA* driven by P*_*exsC*_*

**Figure 9:**
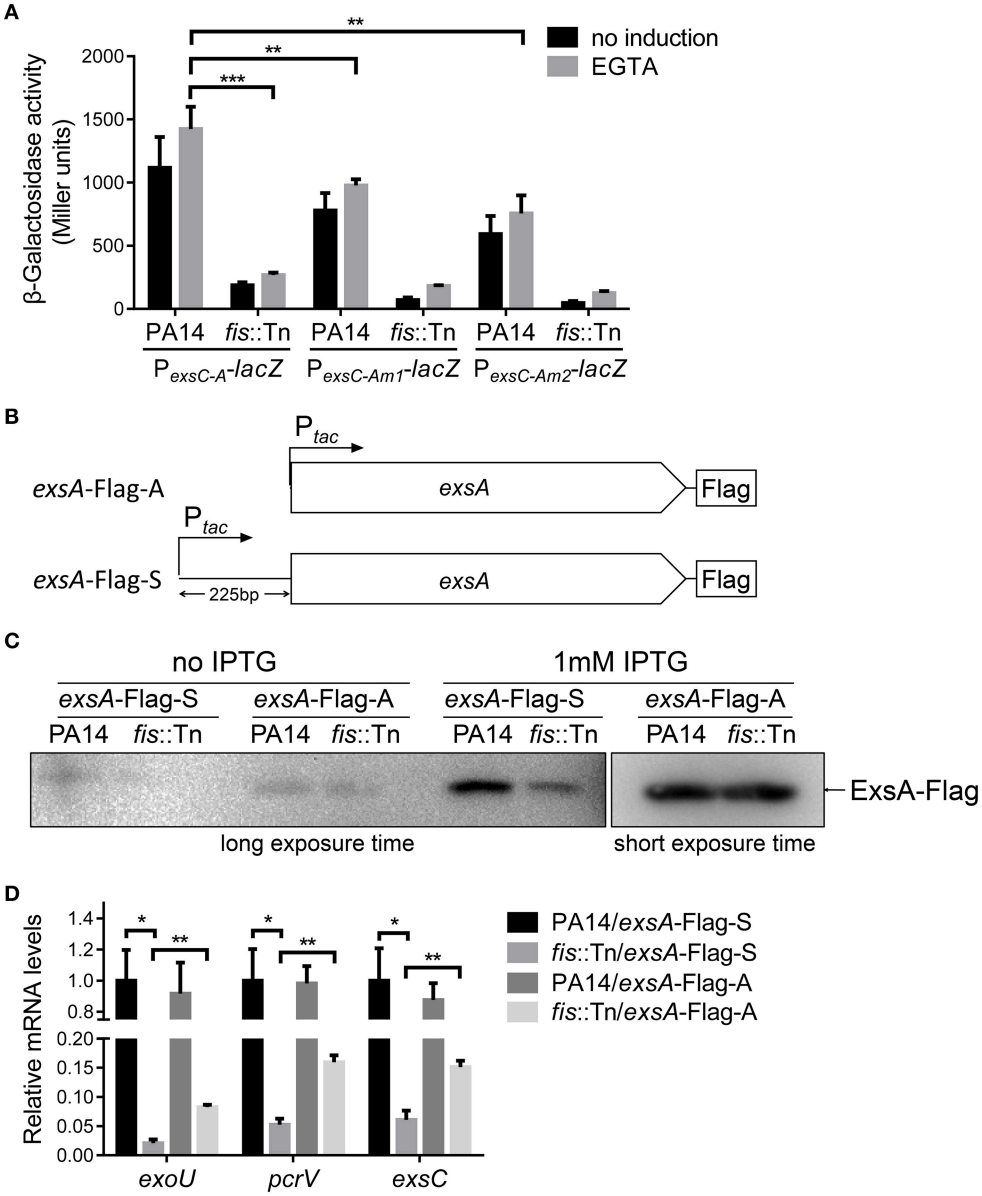
**Role of Fis in the transcription elongation from ***exsB*** to ***exsA***. (A)** PA14 and *fis*::Tn mutant carrying P_*exsC-A*_*-lacZ*, P_*exsC-Am*1_-*lacZ*, or P_*exsC-Am*2_-*lacZ* transcriptional reporters were grown at 37°C with or without 5 mM EGTA. The values are the means of at least three independent experiments. ^***^p < 0.001; ^**^p < 0.01 by Student's *t*-test. **(B)** Constructs of *exsA*-Flag-S and *exsA*-Flag-A. *exsA*-Flag-S contains *exsA* ORF and 225 bp upstream fragment fused with *tac* promoter and *exsA*-Flag-A contains *exsA* ORF only fused with *tac* promoter. **(C)** PA14 and *fis*::Tn mutant carrying plasmids *exsA*-Flag-S or *exsA*-Flag-A were grown with or without 1 mM IPTG for 3 h. ExsA-Flag levels were determined by western blot using an anti-Flag antibody. The amounts of protein in different samples were equal. **(D)** Relative mRNA levels of T3SS genes *exoU, pcrV, exsC, exsA*. Total RNA was isolated from bacteria grown with 1 mM IPTG and relative mRNA levels of these genes were determined by quantitative real-time PCR. Data represents the mean ± standard deviation from three samples. ^*^p < 0.05, ^**^p < 0.01 by Student's *t*-test.

We have demonstrated that the P_*exsA*_ activity in the *fis*::Tn mutant was similar to that in the wild type PA14 (Figure 5B). Consistently, insertion of T0T1 up stream of P_*exsA*_ in the *fis*::Tn mutant resulted in similar mRNA level of *exsA* as that in PA14 with the T0T1 insertion (Figure 8A), confirming that Fis is not involved in the regulation of P_*exsA*_ activity. Thus, we suspected that Fis might affect the transcription of *exsA* driven by P_*exsC*_ and subsequent expression of T3SS genes. To test this possibility, we transferred the P_*exsC-A*_-*lacZ* transcriptional fusion into the *fis*::Tn mutant. The β-galactosidase level was significantly lower in the *fis*::Tn mutant than that in the wild type PA14 (Figure 9A). To further verify that Fis affects the transcription of *exsA* driven by P_*exsC*_ by directly binding to the *exsB*-*exsA* intergenic region, the conserved nucleotides inside the Fis binding sequence were mutated (Figure 6A), which had been shown to significantly reduced the binding between Fis and the fragment (Figure 4C). The mutated P_*exsC-A*_-*lacZ* fusions were designated as P_*exsC-Am*1_-*lacZ* and P_*exsC-Am*2_-*lacZ*, respectively (Figure 6A). Compared to the original P_*exsC-A*_-*lacZ*, the P_*exsC-Am*1_-*lacZ*, and P_*exsC-Am*2_-*lacZ* yielded lower levels of β-galactosidase in wild type PA14 (Figure 9A). In combination, these results suggest that Fis affects the P_*exsC*_ driven *exsA* transcription through direct binding to the *exsB*-*exsA* intergenic region.

We then isolated RNA from the *fis*::Tn mutant under T3SS inducing or non-inducing conditions and performed RT-PCR with the primers annealing to the coding regions of *exsB* and *exsA*. The PCR product level was much lower than that in the wild type PA14 (Figure 7B). Consistently, real time PCR results revealed that the RNA spanning the *exsB-exsA* intergenic region was less in the *fis*::Tn mutant under T3SS inducing condition (Figure 7C). These results suggest a role of Fis in the continuous transcription from *exsB* to *exsA*. Since ExsA activates P_*exsC*_ (Diaz et al., 2011), our observation might be due to defective translation of the ExsA indirectly affected by Fis. To test this possibility, we utilized two previously constructed C-terminus FLAG-tagged *exsA* (*exsA*-Flag) driven by an exogenous *tac* promoter (Li et al., 2013). In one of the construct, namely *exsA*-Flag-A, the *exsA* coding region was directly fused with the *tac* promoter, whereas in the other construct (*exsA*-Flag-S), the *exsB*-*exsA* intergenic region was included (Figure 9B). Without the *exsB*-*exsA* intergenic region, similar levels of ExsA-FLAG were observed in wild type PA14 and the *fis*::Tn mutant. However, presence of the *exsB*-*exsA* intergenic region resulted in less ExsA-FLAG in the *fis*::Tn mutant (Figure 9C), thus confirming the role of Fis in the continuous transcription from *exsB* to *exsA*. Consistently, in the *fis*::Tn mutant the *exsA*-Flag-A resulted in higher mRNA levels of *exoU, exsC*, and *pcrV* than the *exsA*-Flag-S (Figure 9D). In wild type PA14, the *exsA*-Flag-A resulted in higher mRNA levels of *exoU, exsC*, and *pcrV* than those in the *fis*::Tn mutant, which might be due to higher expression of the chromosomal *exsA*. In combination, these results suggest that Fis is required for the P_*exsC*_–dependent transcription of *exsA*, which is required for the full activation of the T3SS.

## Discussion

In this study, we demonstrate that Fis is involved in the regulation of T3SS in *P. aeruginosa*. Since negative supercoiling of DNA can influence transcription elongation (Baaklini et al., 2004; Travers and Muskhelishvili, 2005) and Fis is considered to be a local topological homeostat (Travers and Muskhelishvili, 2005), Fis might be involved in the regulation of transcription elongation. DNA regulated by Fis might require Fis binding and bending in multiple tandem sites in a non-random fashion (Schneider et al., 2001; Kahramanoglou et al., 2011). In addition, Fis displays a preference for binding particularly at regions upstream of open reading frames (Kahramanoglou et al., 2011). For example, the *tyrT* promoter is regulated by three Fis dimers binding to the core and upstream regions of the *tyrT* promoter (Pemberton et al., 2002). Here, we found that Fis specifically interacts with the -10 box of P_*exsA*_ promoter and directly controls mRNA transcription elongation initiated from P_*exsC*_ promoter. However, we cannot eliminate the possibility of additional Fis binding sites upstream or inside the *exsCEBA* operon, i.e., Fis may interact with multiple sites to control the transcription of *exsCEBA*. It has been found that the expression of *exsA* is regulated at the post-transcriptional level (Intile et al., 2015; Tan et al., 2016). Besides, multiple genes have been found to affect the expression of *exsA* (Dong et al., 2013; Marsden et al., 2016; Yu et al., 2016; Zhu et al., 2016a,b). Since Fis functions as a global regulator, it is possible that Fis indirectly regulates ExsA expression at both transcriptional and post-transcriptional level.

ExsA is the master transcriptional activator for the T3SS genes in *P. aeruginosa*, including *exsC* (Diaz et al., 2011). Recently, it has been reported that the transcription of *exsA* is controlled by a Vfr-dependent P_*exsA*_ promoter located in the *exsB*-*exsA* intergenic region (Marsden et al., 2016). Together with our findings in this study, we propose the following regulatory mechanism for the expression of *exsA*. T3SS inducing signals stimulate the increase of intracellular cAMP level (Fuchs et al., 2010). Subsequently, Vfr binds to cAMP and activates P_*exsA*_ (Marsden et al., 2016), resulting in higher amount of ExsA, which activates the P_*exsC*_. With the help of Fis, the transcription from P_*exsC*_ extends into *exsA*, which generates a positive feedback loop, resulting in a quick up regulation of the *exsA*. In this way, bacteria can quickly express T3SS genes in response to environmental stimulations.

Fis is a growth phase dependent global regulator in Enterobacteriaceae (Mallik et al., 2006; Bradley et al., 2007; Cho et al., 2008). In *E. coli*, it has been demonstrated that the level of Fis peaks in the early exponential phase, and drops upon entering the stationary phase (Mallik et al., 2006; Bradley et al., 2007). Meanwhile, Fis also responds to the nutritional changes in Enterobacteriaceae (Mallik et al., 2004). However, the Fis expression pattern is not well known in *P. aeruginosa*. Recently, Lu *et al* identified a small regulatory RNA (sRNA) named RgsA which regulates Fis expression at the post-transcriptional level in *P. aeruginosa* (Lu et al., 2016). RgsA is directly controlled by sigma factor σ^S^ (RpoS) (Lu et al., 2016). It is well known that RpoS is up regulated when bacteria enter into stationary phase, and plays crucial role in bacterial survival in stationary phase or under various stress conditions (Hengge-Aronis, 2002). So we suspect that when bacteria enter the stationary phase, the increased level of RpoS leads to up regulation of RgsA. Subsequently, RgsA represses Fis expression at the post-transcriptional level. The expression pattern of the Fis in *P. aeruginosa* warrants further studies. While growing the bacteria, we found that the *fis*::Tn mutant produced higher amount of pyocyanin at the stationary growth phase (data not shown). These results imply a role of Fis in the quorum sensing system. In addition, RgsA is indirectly regulated by the GacS/GacA two-component system (González et al., 2008), which has been demonstrated to regulate T3SS through small RNAs RsmY and RsmZ (Vakulskas et al., 2009; Chen et al., 2016). Therefore, RgsA and Fis may also participate in the T3SS regulation via GacS/GacA.

Previous studies in various bacteria demonstrate that Fis plays pleiotropic roles in bacterial virulence and response to environmental stresses (Duprey et al., 2014), however, the role of Fis in *P. aeruginosa* remains largely unknown. Previous studies demonstrated that Fis is required for swarming motility in *P. aeruginosa* (Yeung et al., 2009; Lu et al., 2016). Other than T3SS, our transcriptome analysis revealed that pyocin biosynthesis genes were up regulated in the *fis*::Tn mutant (Table S2). Biosynthesis of pyocins has been demonstrated to correlate to bacterial susceptibility to DNA damaging agents (Chen et al., 2017). Indeed, we found that the *fis*::Tn mutant was more susceptible to quinolone antibiotics such as ciprofloxacin and ofloxacin (data not shown). Further studies are needed to examine the role of Fis in the regulation of pyocin biosynthesis and whether abnormal expression of pyocin genes contributes to the increased susceptibility in the *fis*::Tn mutant (Agnello et al., 2016). In addition, although overexpression of ExsA in the *fis*::Tn mutant fully restored the bacterial cytotoxicity, the bacterial virulence was partially restored, indicating additional virulence factors might be regulated by Fis. Therefore, genes directly regulated by Fis as well as the global binding site of Fis in *P. aeruginosa* warrants further studies.

## Author contributions

Conceived and designed the experiments and wrote the paper: WW, XD, ZC, and SJ. Performed the experiments: XD, ML, XP, RZ, CL, FC, and XL. Analyzed the data: XD, ML, WW, ZC, and SJ.

## Funding

This work was supported by National Science Foundation of China (31670130, 31370168, 31370167, and 31600110); Program of international S&T cooperation (2015DFG32500) and Science and Technology Committee of Tianjin (15JCYBJC53900 and 15JCZDJC33000). The funders had no role in study design, data collection and interpretation, or the decision to submit the work for publication.

### Conflict of interest statement

The authors declare that the research was conducted in the absence of any commercial or financial relationships that could be construed as a potential conflict of interest.

## Supplementary material

Figure S1 **Relative mRNA level of PA4852**. Total RNA was isolated from bacteria at indicated optical densities (OD_600_). cDNA was synthesized with PA4852 and *rpsL* specific primer. Relative mRNA levels of PA4852 were determined by quantitative real-time PCR. *rpsL* was used as an internal control. Data represents the mean ± standard deviation from three samples.

Figure S2 **Relative mRNA level of RgsA**. Total RNA was isolated from bacteria at indicated optical densities (OD_600_). Relative mRNA levels of RgsA were determined by quantitative real-time PCR. *rpsL* was used as an internal control. Data represents the mean ± standard deviation from three samples.

Figure S3 *****exsC*** promoter activity**. PA14 and the *fis*::Tn mutant carrying P_*exsC*_*-lacZ* were grown at 37°C with or without 5 mM EGTA and assayed for β-galactosidase activities. The reported values (Miller units) are the means of results of at least three independent experiments. ^***^*p* < 0.001 by Student's *t*-test.

Figure S4 **Growth curves of the ***fis***::Tn mutant and the wild type PA14**. Indicated strains were grown at 37°C in LB medium. Bacterial numbers were determined at indicated time by serial dilution and plating. The error bars indicate standard error of the mean.

Figure S5 **Growth curves of the wild type PA14, ***fis***::Tn mutant and the ***exsA*** overexpression strain**. Indicated strains were grown at 37°C in LB medium. Bacterial numbers were determined at indicated time by serial dilution and plating. The error bars indicate standard error of the mean.

Figure S6 **EMSA for Fis binding to the P_***exsC***_ promoter**. Fis was incubated with P_*exsC*_ promoter probes i, ii, iii for 30 min at 25°C. Probes i, ii, iii represent the upstream region of P_*exsC*_ -35 box, P_*exsC*_ promoter -35 box, and P_*exsC*_ promoter -10 box, respectively. Arrows indicate the positions of unbound probes and LMC.

Table S1 **Bacterial strains, plasmids, and primers used in this study**.

Table S2 **PA14 Transcriptome analysis: differentially expressed genes**.
