# Supplementary figures and images for: Fis Regulates Type III Secretion System by Influencing the Transcription of exsA in Pseudomonas aeruginosa Strain PA14
Source: Front Microbiol. 2017 Apr 19;8:669. doi: 10.3389/fmicb.2017.00669 (PMC5395579; doi:10.3389/fmicb.2017.00669)

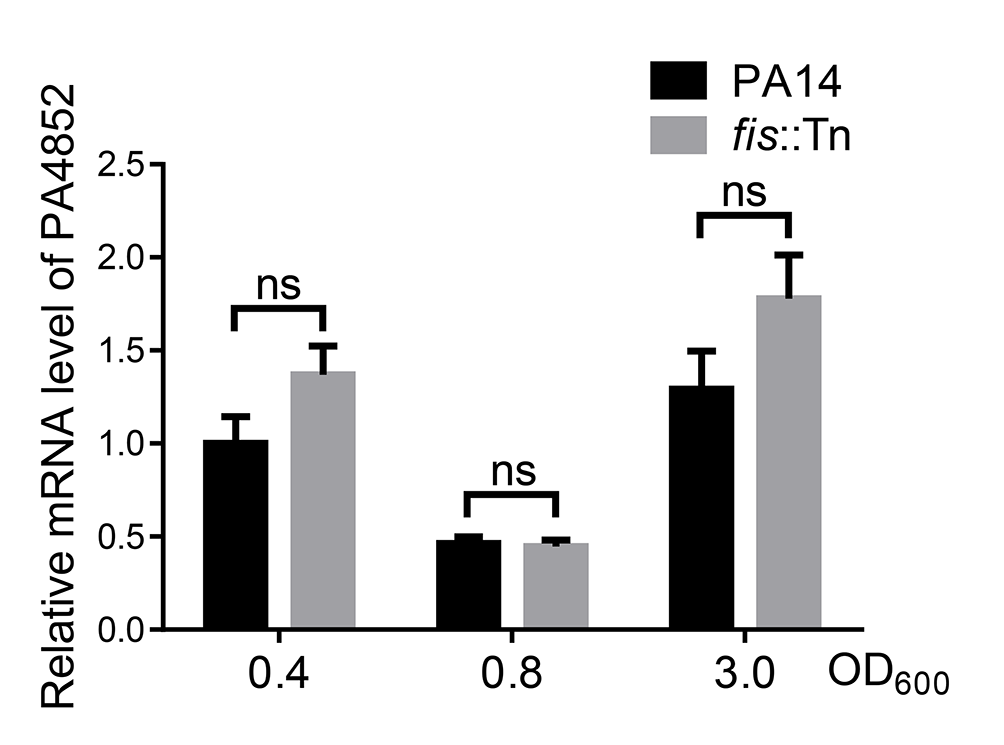

Supplement: Figure S1 — Relative mRNA level of PA4852. Total RNA was isolated from bacteria at indicated optical densities (OD600). cDNA was synthesized with PA4852 and rpsL specific primer. Relative mRNA levels of PA4852 were determined by quantitative real-time PCR. rpsL was used as an internal control. Data represents the mean ± standard deviation from three samples. [file Image1.TIF]

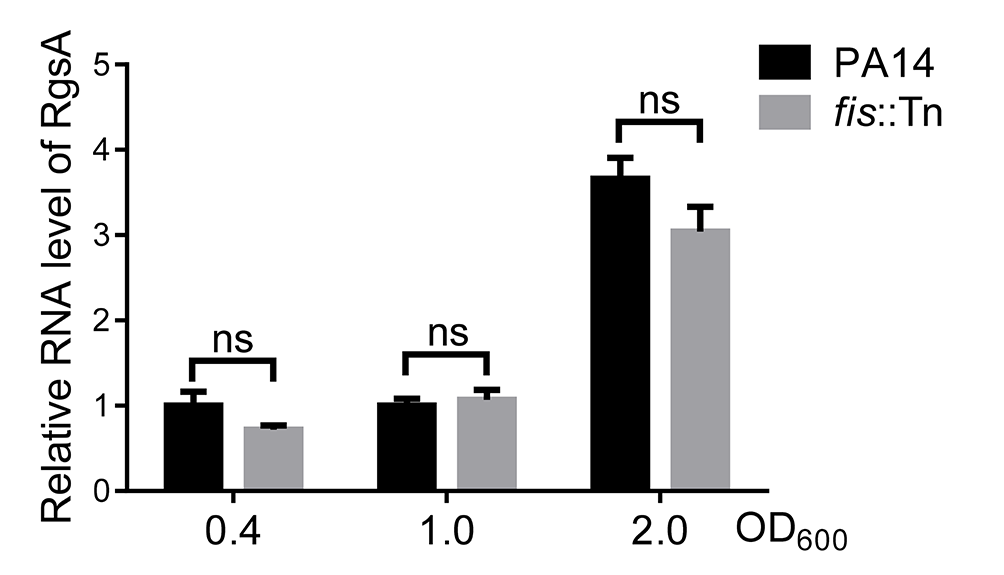

Supplement: Figure S2 — Relative mRNA level of RgsA. Total RNA was isolated from bacteria at indicated optical densities (OD600). Relative mRNA levels of RgsA were determined by quantitative real-time PCR. rpsL was used as an internal control. Data represents the mean ± standard deviation from three samples. [file Image2.TIF]

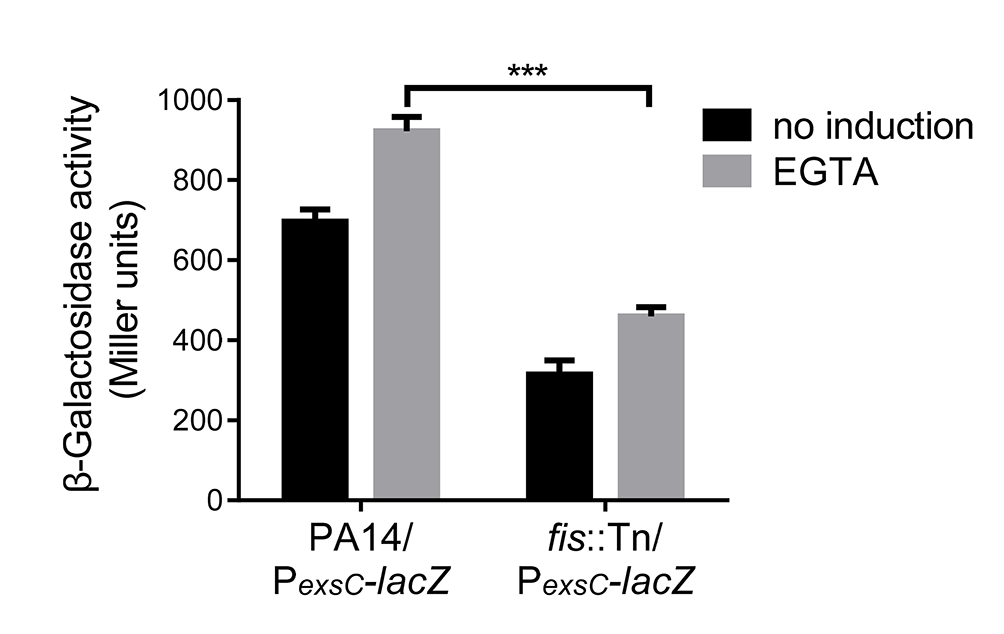

Supplement: Figure S3 — exsC promoter activity. PA14 and the fis::Tn mutant carrying PexsC-lacZ were grown at 37°C with or without 5 mM EGTA and assayed for β-galactosidase activities. The reported values (Miller units) are the means of results of at least three independent experiments. ***p < 0.001 by Student's t-test. [file Image3.TIF]

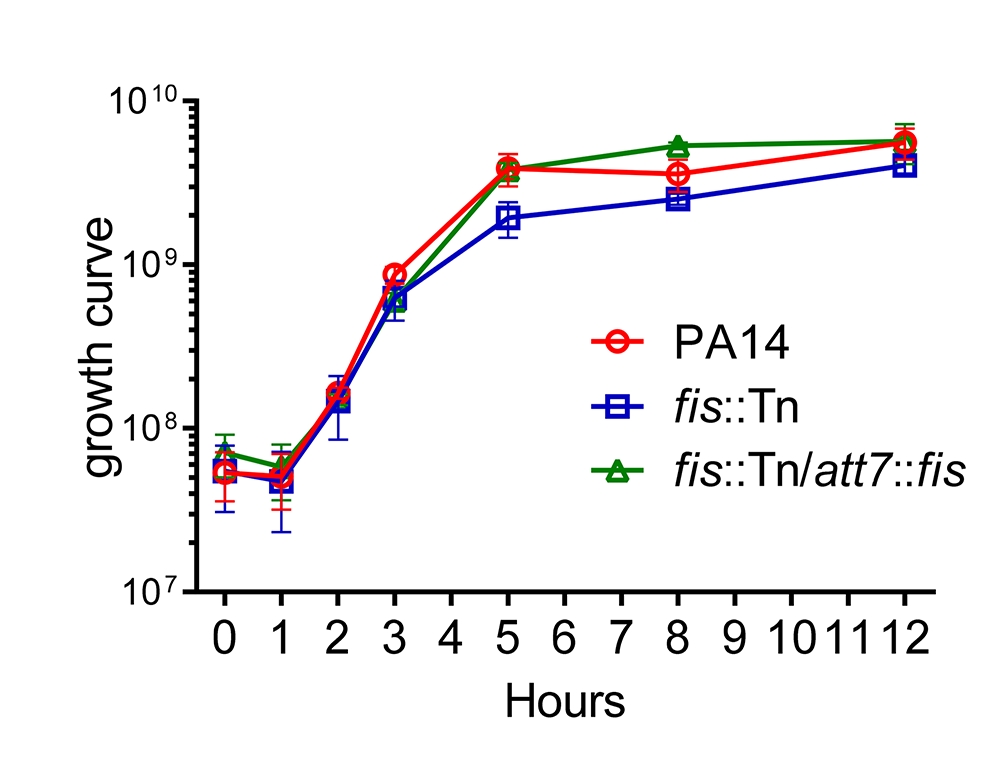

Supplement: Figure S4 — Growth curves of the fis::Tn mutant and the wild type PA14. Indicated strains were grown at 37°C in LB medium. Bacterial numbers were determined at indicated time by serial dilution and plating. The error bars indicate standard error of the mean. [file Image4.TIF]

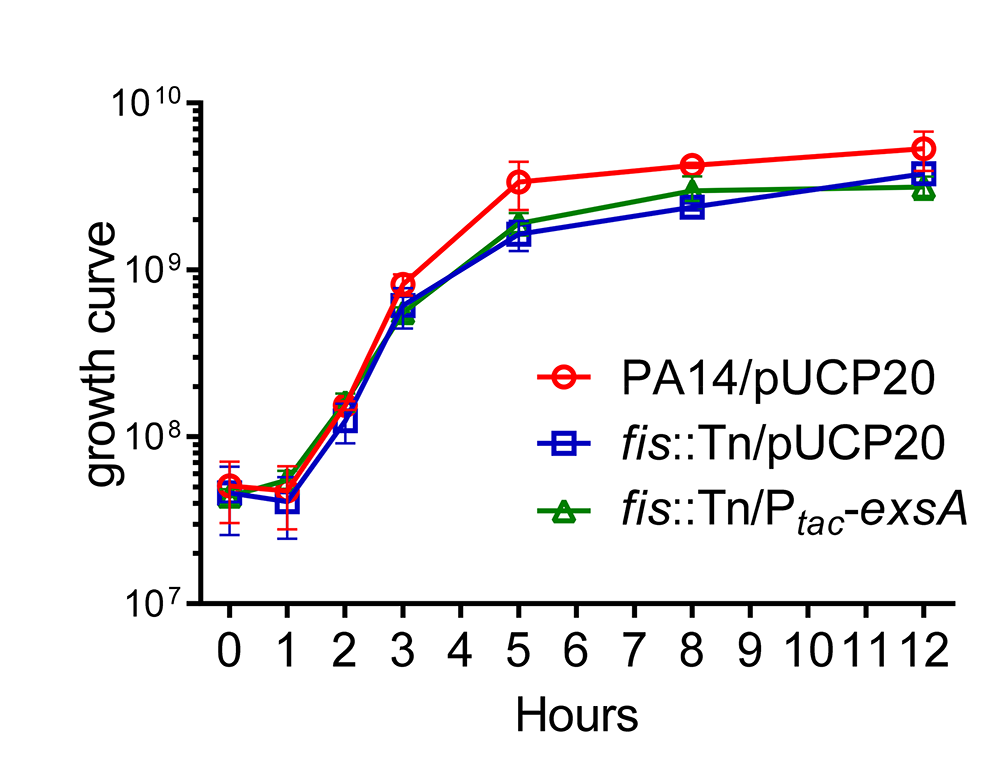

Supplement: Figure S5 — Growth curves of the wild type PA14, fis::Tn mutant and the exsA overexpression strain. Indicated strains were grown at 37°C in LB medium. Bacterial numbers were determined at indicated time by serial dilution and plating. The error bars indicate standard error of the mean. [file Image5.TIF]

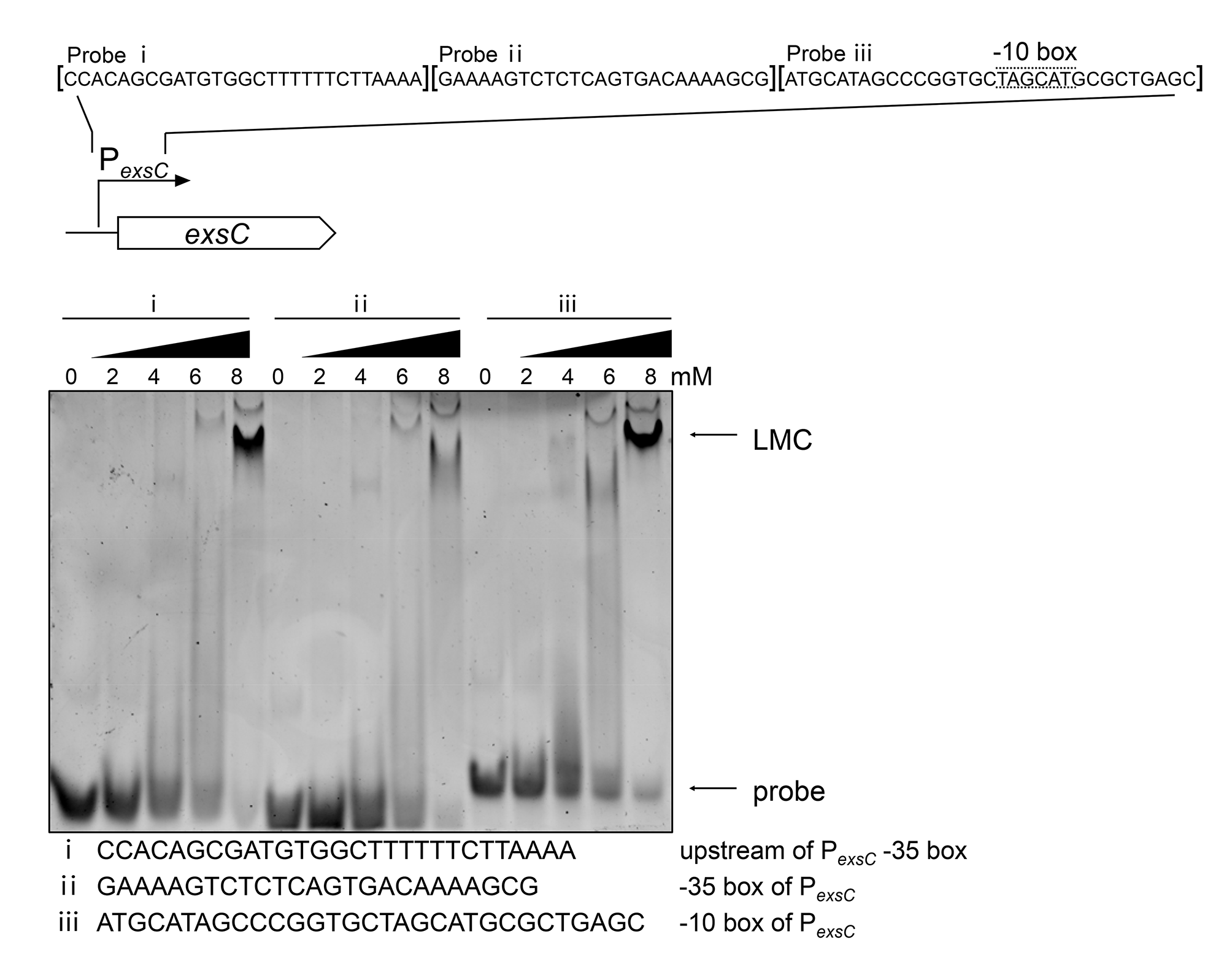

Supplement: Figure S6 — EMSA for Fis binding to the PexsC promoter. Fis was incubated with PexsC promoter probes i, ii, iii for 30 min at 25°C. Probes i, ii, iii represent the upstream region of PexsC -35 box, PexsC promoter -35 box, and PexsC promoter -10 box, respectively. Arrows indicate the positions of unbound probes and LMC. [file Image6.TIF]
