# Supplementary material for: Fis Regulates Type III Secretion System by Influencing the Transcription of exsA in Pseudomonas aeruginosa Strain PA14
Source: Front Microbiol. 2017 Apr 19;8:669. doi: 10.3389/fmicb.2017.00669 (PMC5395579; doi:10.3389/fmicb.2017.00669)
Supplement: Table S1 — Bacterial strains, plasmids, and primers used in this study. [file Table1.DOCX]

Table S1. Bacterial strains, plasmids and primers used in this study.

| Strain/ Plasmid/ Primer | Description | Source (Reference) |
| --- | --- | --- |
| **Strains**  ***E. coli***  DH5α  S17-1  ***P. aeruginosa***  PA14  *fis*::Tn  *fis*::Tn/*att7*::*fis*  Δ*fis*/pMMB67EH-*fis*-His  *fis*::Tn/pUCP20-P*_tac_*-*exsA*  PA14 T0T1  *fis*::Tn T0T1  **Plasmid**  pEX18Tc  pUC18T-mini-Tn7T-Tc  pUC18T-mini-Tn7T-Tc-*fis*  pMMB67EH  pMMB67EH-*fis*-His  pUCP20-P*_tac_*-*exsA*  pEX18Tc-T0T1 insertion  *exsA*-Flag-A  *exsA*-Flag-S  pDN19lacΩ  P*_exsA_*-*lacZ*  P*_exsC_*-*lacZ*  P*_exsC-A_*-*lacZ*  P*_exsC-Am1_*-*lacZ*  P*_exsC-Am2_*-*lacZ*  pUCP20-P*_exsA_*-*exsA*-His  pUCP20-P*_exoU_*-*exoU*-His  **Primer**  PA4853pup  PA4853pdown  PA4853up  PA4853down  PA4853sense2  PA4853antisense2  PA4852SENSE  PA4852ANTISENSE  exsC sense  exsC antisense  exsU sense  exsU antisense  exsA’ sense  exsA’ antisense  PexsA fis up2  PexsA-flag-his  B+A UP  B+A DOWN  PexsC-Aup  PexsC-Adown  T0T1upstreamF  T0T1upstreamR  T0T1downstreamF  T0T1downstreamR  T0T1up  T0T1down  Overlap shang m1  Overlap xia m1  Overlap shang m2  Overlap xia m2 | F^-^, φ80d*lacZ*ΔM15, Δ(*lacZYA-argF*)U169, *deoR*, *recA1*, *endA1*, *hsdR17*(*r*_k_^-^,*m*_k_^+^), *phoA*, *supE44*, λ^-^, *thi-1*, *gyrA96*, *relA1*  *recA*, *pro*, *hsdR*, RP4-2-Tc::Mu-Km::Tn7  Wild type strain of *Pseudomonas aeruginosa*  PA14 with MAR2xT7 transposon inserted at *fis*; Gm^r^  *fis*::Tn with *fis* inserted on chromosome with mini-Tn7T insertion; Gm^r^, Tc^r^  PA14 knockout *fis* with *fis*-His is driven by an inducible *tac* promoter; Cb^r^  *fis*::Tn with *exsA* overexpression  PA14 with terminators T0T1 inserted at *exsB*-*exsA* intergenic region before *exsA* promoter  *fis*::Tn with terminators T0T1 inserted at *exsB*-*exsA* intergenic region before *exsA* promoter; Gm^r^  Gene replacement vector; Tc^r^, *oriT*^+^, *sacB*^+^  mini-Tn7 base vector from insertion into chromosome attTn7 site; Tc^r^  pUC18T-mini-Tn7T-Tc with *fis*; Tc^r^  Expression vector with *tac* promoter; Ap^r^  pMMB67EH with *fis*-His driven by an inducible *tac* promoter; Ap^r^  pUCP20 with *exsA* driven by *tac* promoter  T0T1 insertion at PA14 *exsB*-*exsA* intergenic region on pEX18Tc; Tc^r^  *exsA*-Flag-CTC (containing *exsA* ORF only) fused with pDN19; Ap^r^ , Tc^r^  *exsA*-Flag-CTC (containing *exsA* ORF and 225bp upstream fragment) fused with pDN19; Ap^r^ , Tc^r^  Promoterless lacZ fusion vector, Sp^r^, Sm^r^, Tc^r^  *exsA* promoter-*lacZ* fusion reporter in pDN19lacΩ; Sp^r^, Sm^r^, Tc^r^  *exsC* promoter*-lacZ* fusion reporter in pDN19lacΩ; Sp^r^, Sm^r^, Tc^r^  *exsC* promoter to *exsA* promoter*-lacZ* fusion reporter in pDN19lacΩ; Sp^r^, Sm^r^, Tc^r^  points mutation in P*_exsC-A_*-*lacZ*; Sp^r^, Sm^r^, Tc^r^  points mutation in P*_exsC-A_*-*lacZ*; Sp^r^, Sm^r^, Tc^r^  *exsA* promoter of PA14 fused to *exsA-His* on promoterless pUCP20; Ap^r^  *exoU* promoter of PA14 fused to *exoU-His* on promoterless pUCP20; Ap^r^  **Sequence (5’→3’)**  CGCGGATCCGGTCCGCAGCCATCCCGAATTCAGC  AAAACTGCAGGGGGCTTCCCTGTCTTGCGGTGCTG  AAAACTGCAGACAACGGAACAGGGGTGGCCGCATG  CCGGAATTCGGAGCCCGCCGCCGCTCTTAAAGAA  TTACAACATGGTGCTCTG  ATCGTATTGCTTGAGTTTCT  CAATGTGGAACTCGGTGC  GCTGCCTTGTTGCATACC  ATGGATTTAACGAGCAAGGTCAA  GAGGGACAGGGAAGGCAAA  CTTCAGAGCGTCATACCT  CAACACTGGTGAGCATAC  AAGGAGCCAAATCTCTTG  CTTGTTTACCCTGTATTCG  GCTCTAGATGATACATTGCCTGCT  CCCAAGCTTTCAGTGGTGGTGGTGGTGGTGCTTATCGTCGTCATC  TCGCCCGGAAGAAAGATCTGGC  CCCTGTATTCGAAAGTTGGAATGT  CCGGAATTCGGTGATCCAGTCCTTCGTCCAGATG  CGCGGATCCTTATAAGAACCCCAACACTTCCCGTC  CCGGAATTCGCACCGTTTCGATCTGCATTTC  CGCGGATCCCGAGACTTGCACTTCTTTAATCTCCATA  CTAGTCTAGAGTTCGTTGTCAGGGAAGGCCTCG  CCCAAGCTTACTGACTGGAAAAGCCCGCCTC  CGCGGATCCGACTCCTGTTGATAGATCCAGTAATGACCTC  CTAGTCTAGAGGCGGATTTGTCCTACTCAGGAGAG  CACGGAGTCCATTTTATAATAATGAGGATTATC  GATAATCCTCATTATTATAAAATGGACTCCGTG  CACGGAGTCGATATTATAATTATCAGGATTATC  GATAATCCTGATAATTATAATATCGACTCCGTG | TransGen  ([Simon et al., 1983](#_ENREF_7))  ([Liberati et al., 2006](#_ENREF_6))  ([Liberati et al., 2006](#_ENREF_6))  this study  this study  this study  this study  this study  ([Hoang et al., 1998](#_ENREF_3))  ([Choi and Schweizer, 2006](#_ENREF_1))  this study  ([Furste et al., 1986](#_ENREF_2))  this study  this study  this study  ([Li et al., 2013](#_ENREF_4))  ([Li et al., 2013](#_ENREF_4))  ([Totten and Lory, 1990](#_ENREF_8))  this study  this study  this study  this study  this study  this study  ([Li et al., 2016](#_ENREF_5))  **Purpose**  *fis* promoter cloning  *fis* promoter cloning  *fis* cloning  *fis* cloning  real-time PCR  real-time PCR  real-time PCR  real-time PCR  real-time PCR  real-time PCR  real-time PCR  real-time PCR  real-time PCR  real-time PCR  *exsA* cloning  *exsA* cloning  RT-PCR  RT-PCR  *exsC-A* promoter cloning  *exsC-A* promoter cloning  T0T1 insertion  T0T1 insertion  T0T1 insertion  T0T1 insertion  T0T1 insertion  T0T1 insertion  site-directed mutagenesis  site-directed mutagenesis  site-directed mutagenesis  site-directed mutagenesis |

Choi, K.H., and Schweizer, H.P. (2006). mini-Tn7 insertion in bacteria with single attTn7 sites: example Pseudomonas aeruginosa. *Nat Protoc* 1(1)**,** 153-161. doi: 10.1038/nprot.2006.24.

Furste, J.P., Pansegrau, W., Frank, R., Blocker, H., Scholz, P., Bagdasarian, M., et al. (1986). Molecular cloning of the plasmid RP4 primase region in a multi-host-range tacP expression vector. *Gene* 48(1)**,** 119-131.

Hoang, T.T., Karkhoff-Schweizer, R.R., Kutchma, A.J., and Schweizer, H.P. (1998). A broad-host-range Flp-FRT recombination system for site-specific excision of chromosomally-located DNA sequences: application for isolation of unmarked Pseudomonas aeruginosa mutants. *Gene* 212(1)**,** 77-86.

Li, K., Xu, C., Jin, Y., Sun, Z., Liu, C., Shi, J., et al. (2013). SuhB is a regulator of multiple virulence genes and essential for pathogenesis of Pseudomonas aeruginosa. *MBio* 4(6)**,** e00419-00413. doi: 10.1128/mBio.00419-13.

Li, M., Long, Y., Liu, Y., Liu, Y., Chen, R., Shi, J., et al. (2016). HigB of Pseudomonas aeruginosa Enhances Killing of Phagocytes by Up-Regulating the Type III Secretion System in Ciprofloxacin Induced Persister Cells. *Front Cell Infect Microbiol* 6**,** 125. doi: 10.3389/fcimb.2016.00125.

Liberati, N.T., Urbach, J.M., Miyata, S., Lee, D.G., Drenkard, E., Wu, G., et al. (2006). An ordered, nonredundant library of Pseudomonas aeruginosa strain PA14 transposon insertion mutants. *Proc Natl Acad Sci U S A* 103(8)**,** 2833-2838. doi: 10.1073/pnas.0511100103.

Simon, R., Priefer, U., and Pühler, A. (1983). A Broad Host Range Mobilization System for In Vivo Genetic Engineering: Transposon Mutagenesis in Gram Negative Bacteria. *Nature Biotechnology* 1(9)**,** 784-791.

Totten, P.A., and Lory, S. (1990). Characterization of the type a flagellin gene from Pseudomonas aeruginosa PAK. *J Bacteriol* 172(12)**,** 7188-7199.
